# Synergistic Induction of Potential Warburg Effect in Zebrafish Hepatocellular Carcinoma by Co-Transgenic Expression of *Myc* and *xmrk* Oncogenes

**DOI:** 10.1371/journal.pone.0132319

**Published:** 2015-07-06

**Authors:** Zhen Li, Weiling Zheng, Hankun Li, Caixia Li, Zhiyuan Gong

**Affiliations:** Department of Biological Sciences, National University of Singapore, 117543, Singapore, Singapore; Institute of Cellular and Organismic Biology, TAIWAN

## Abstract

Previously we have generated inducible liver tumor models by transgenic expression of *Myc* or *xmrk* (activated *EGFR* homolog) oncogenes in zebrafish. To investigate the interaction of the two oncogenes, we crossed the two transgenic lines and observed more severe and faster hepatocarcinogenesis in *Myc*/*xmrk* double transgenic zebrafish than either single transgenic fish. RNA-Seq analyses revealed distinct changes in many molecular pathways among the three types of liver tumors. In particular, we found dramatic alteration of cancer metabolism based on the uniquely enriched pathways in the *Myc/xmrk* tumors. Critical glycolytic genes including *hk2*, *pkm* and *ldha* were significantly up-regulated in *Myc/xmrk* tumors but not in either single oncogene-induced tumors, suggesting a potential Warburg effect. In RT-qPCR analyses, the specific *pkm2* isoformin Warburg effect was found to be highly enriched in the *Myc/xmrk* tumors but not in *Myc* or *xmrk* tumors, consistent with the observations in many human cancers with Warburg effect. Moreover, the splicing factor genes (*hnrnpa1*, *ptbp1a*, *ptbp1b and sfrs3b*) responsible for generating the *pkm* isoform were also greatly up-regulated in the *Myc/xmrk* tumors. As Pkm2 isoform is generally inactive and causes incomplete glycolysis to favor anabolism and tumor growth, by treatment with a Pkm2-specific activator, TEPP-46, we further demonstrated that activation of Pkm2 suppressed the growth of oncogenic liver as well as proliferation of liver cells. Collectively, our *Myc/xmrk* zebrafish model suggests synergetic effect of EGFR and MYC in triggering Warburg effect in the HCC formation and may provide a promising *in vivo* model for Warburg effect.

## Introduction

HCC is currently the third leading cause of cancer-related death and has an increasing trend in recent years[1]. The prognosis of HCC remains dismal with the median survival rates of <2 years from diagnosis and of <5 months without effective treatment [2].Pathogenesis of liver cancer is highly complex due to different etiology and deregulation of various signaling pathways. Nevertheless, it has been demonstrated that manipulation of one critical signaling component or biological pathway is sufficient to cause hepatocarcinogenesis [3, 4]. The dependence of a single oncogene to maintain tumor state has been proved in many animal models [5, 6], including our recent liver tumor transgenic zebrafish model [7–9]. Furthermore, it has also been documented that co-expression of two oncogenes in the mouse liver could accelerate hepatocarcinogenesis and result in more severe phenotype, including the co-expression of Myc with SV40 T-antigen, H-ras, TGFα or E2F1 [10–14]. However, the molecular mechanisms of the synergetic effect, which likely occur frequently in clinical tumors, remain unelucidated.

*MYC* is frequently amplified in human HCC and associated with unfavorable prognosis[15]. MYC is known to regulate a wide spectrum of tumorigenesis-related cell behaviors, including proliferation, survival, differentiation, and genetic stability [16]. Particularly, MYC has been shown to directly stimulate ribosome biogenesis by regulating the transcriptional control of RNA and protein components of ribosomes, rRNA processing, nuclear export of ribosomal subunits, and the initiation of mRNA translation [17]. It has been suggested in many cases that the ability of MYC to initiate tumorigenesis is related to its ability to regulate ribosome biogenesis [18–20]. Furthermore, Myc could stimulate nucleus-encoded mitochondrial genes and promote mitochondrial biogenesis [21], which confers a growth advantage to tumor cells [22].

EGFR is commonly up-regulated in human HCC, particularly in advanced HCCs with high proliferating activity, presence of intrahepatic metastasis and poor disease-free survival [23]. Moreover, EGFR has emerged as a signaling hub for various inflammatory signals including growth factors, cytokines, and inflammatory mediators in HCC [24]. EGFR could activate downstream pathways, including RAF/MEK/ERK, PI3K/AKT, and mTOR pathways, thus leading to the activation of survival and proliferation mechanisms [25]. However, the collaborative interaction of Myc and EGFR pathways in hepatocarcinogenesis has not been report, but our previous comparative transcriptomic analyses have indicated that a portion of human HCCs do shown molecular signatures of co-expression of MYC and EGFR [26].

Previously, we have established two transgenic zebrafish models for liver tumors by inducible expression of mouse *Myc* [27] and fish *xmrk* (*Xiphophorus* melanoma receptor kinase) encoding a naturally occurred activated form of EGFR [7], respectively. In order to investigate the interaction of the two oncogenes in hepatocarcinogenesis, here we generated *Myc* and *xmrk* double transgenic zebrafish by crossing the two transgenic lines and found faster hepatocarcinogenesis in the double transgenic fish, indicating a synergetic effect of the two oncogenes in hepatocarcinogenesis. Transcriptomic analyses of the liver tumors from the double transgenic fish indicated that pathways that were up-regulated by both *Myc* and *xmrk* were further up-regulated and pathways differentially regulated by *Myc* and *xmrk* were counterbalanced. Interestingly, the double transgenic fish showed uniquely elevated Warburg effect as compared to either single transgenic fish, indicating that these two intracellular signallings may synergistically trigger the Warburg effect in HCC.

## Material and Methods

### Zebrafish maintenance and induction of liver tumors in transgenic zebrafish

This study involving zebrafish was carried out in strict accordance with the recommendations in the Guide for the Care and Use of Laboratory Animals of the National Institutes of Health. The protocol was approved by the Institutional Animal Care and Use Committee (IACUC) of the National University of Singapore (Protocol Number: 096/12). *Xmrk* transgenic zebrafish, *Tg(fabp10*:*TA; TRE*:*xmrk; krt4*:*GFP)* or *TO(xmrk)*, and *Myc* transgenic zebrafish, *Tg(fabp10*:*TA; TRE*:*Myc; krt4*:*GFP)* or *TO(Myc)*, have been previously described [7, 27]. Heterozygous *TO(xmrk)* were crossed with heterozygous *TO(Myc)* to generate the double transgenic fish TO(Myc/xmrk). Liver tumors were induced by doxycycline treatment to activate oncogenes. Juvenile treatment was started from 21 dpf (day postfertilization) with 30 μg/ml doxycycline while adult male fish were treated with 60 μg/ml doxycycline from 3.5 mpf (month postfertilization). For survival rate monitoring at both juvenile and adult stages, each group started with 40 fish. Adult double-transgenic fish liver samples were collected at 4 wpi (week post-induction) and the other groups of liver samples were collected at 6 wpi. 4–5 fish livers from the same group were pooled to generate one RNA sample for RNA-Seq. Throughout the experiments, humane endpoints were used and the experimental fish were euthanized with 250 mg/L MS222 prior to the defined experimental endpoints. The health of the fish was monitored twice a day and water quality was monitored daily. Euthanasia was conducted when fish showed signs of unrelieved sickness, pain and distress (e.g. inactive, unbalanced swimming; lack of appetite; bleeding from the gill cover and/or change of skin color, etc.).

### Paraffin sectioning and histological analysis

Fish samples were fixed in either Bouin’s fixative or formalin solution (Sigma-Aldrich). Sections at 5-μm thickness were processed using the Reichert-Jung 2030 BIOCUT Microtome and stained with Mayer's Hematoxylin and Eosin.

### Sample collection and RNA-Seq

Total RNA were extracted from control and tumor livers by TRIZOL (Invitrogen).The construction of SAGE (serial analysis of gene expression) libraries and next generation sequencing were performed using SOLiD Analyzer 4 (Applied Biosystems) by Mission Biotech Co. Ltd, Taiwan according to manufacturer’s protocol (Applied Biosystems SOLiD SAGE Guide). Briefly, mRNA was purified using Dynabeads Oligo(dT) EcoP (Invitrogen) and subjected to cDNA synthesis. Synthesized cDNA was digested by NlaIII and EcoP15I, and sequencing adapters were added to the cDNA fragments after each digestion. A total of eight SAGE libraries were sequenced: X-M-D-, X+M-D-, X-M+D-, X+M+D-, X-M-D+, X+M-D+, X-M+D+, and X+M+D+, where X for *xmrk*, M for *Myc* and D for doxycycline treatment.

### GSEA (Gene Get Enrichment Analysis), IPA (Ingenuity Pathway Analysis) and DAVIS (Database for Annotation, Visualization and Integrated Discovery) analyses

GSEA pre-ranked option was used to identify the differentially expressed pathways in the three transgenic zebrafish liver cancers. Briefly, the gene symbols of human homologs of the zebrafish Unigene clusters were ranked using logarithm transformed p-value (base 2). Up-regulated genes were given positive values and down-regulated genes were given negative values. The number of permutation used was 1000. Pathways with nominal p-value <0.05 and FDR (false discovery rate) q-value (FDR) <0.25 were considered statistically significant. IPA was carried out for significantly differentially expressed genes (Ingenuity Systems, www.ingenuity.com/). Core analysis was performed for *xmrk*, *myc* and double transgenic tumor respectively. Biological pathway analyses were also performed for uniquely up- or down-regulated genes in *Myc/xmrk* tumors by using the DAVID online software (http://david.abcc.ncifcrf.gov/).

### RT-qPCR

Total RNA were reverse-transcribed to cDNA using Transcriptor First Strand cDNA Synthesis Kit (Roche).RT-qPCR was performed with cDNA as the template by using the LightCycler 480 SYBR Green I Master system (Roche). Reactions were conducted in triplicate for each sample and primer sequences are:*pkm1* (*pkm202*), 5’-CGGAGAGACCGCTAAAGGAGAT (forward) and 5’-CCGGACCCAGTGAGCACTATAA (reverse); *pkm2* (*pkm201*), 5’-AGTGATGTGGCCAATGCAGTTC (forward) and 5’-CAGCATTTGAAGGAAGCCTCGAC (reverse); *pklr*, 5’-CACTCATCAGTATCACGCAGAGAC (forward) and 5’-GGGATAATCCATCCAGATCACGG (reverse); β*-actin*, 5’-CGGTGACATCAAGGAGAAGCT (forward) and 5’-TCGTGGATACCGCAAGATTCC (reverse).Gene expression levels were normalized with the levels of β-actin mRNA.Log2 fold changes between tumour and control samples were calculated using the Ct method according to the formula: log2 fold changes = -ΔΔCt = -[(Ct gene of interest-Ct β-actin) transgenic sample-(Ct gene of interest-Ct β-actin) control sample].

### TEPP-46 treatment and proliferation assay

TEPP-46 was purchased from Cayman Chemical, USA. 10 μM TEPP-46 was used to treat zebrafish larvae from 4 dpf for for 96 hours. In all experimental groups, the survival rates at the end of experiment were always above 90%. After 96 hours treatment, images were taken and liver sizes were measured based on 2D images by using ImageJ (1.49j) as previously described [28, 29]. The average liver sizes were calculated and summarized by using Graphpad Prism 6. Proliferation assay was performed by immunohistochemical staining on cryosections with anti-PCNA (Anaspec) as the primary antibody as previously described [7]. Images were taken for stained sections, and the number of total liver cells and proliferating cells were counted by using ImageJ (1.49j).

### Statistical analyses

Statistical significance between two groups was evaluated by two-tailed unpaired Student t-test using inStat version 5.0 for Windows (GraphPad, San Diego, CA). Statistical data are presented as mean value±standard error of mean (SEM). Throughout the text, figures, and figure legends, the following terminology is used to denote statistical significance: *P<0.05, **P<0.01, ***P<0.001, ****P<0.0001.

## Results

### More severe liver cancer phenotype induced from *Myc* and *xmrk* Double Transgenic Fish

**Fig 1:**
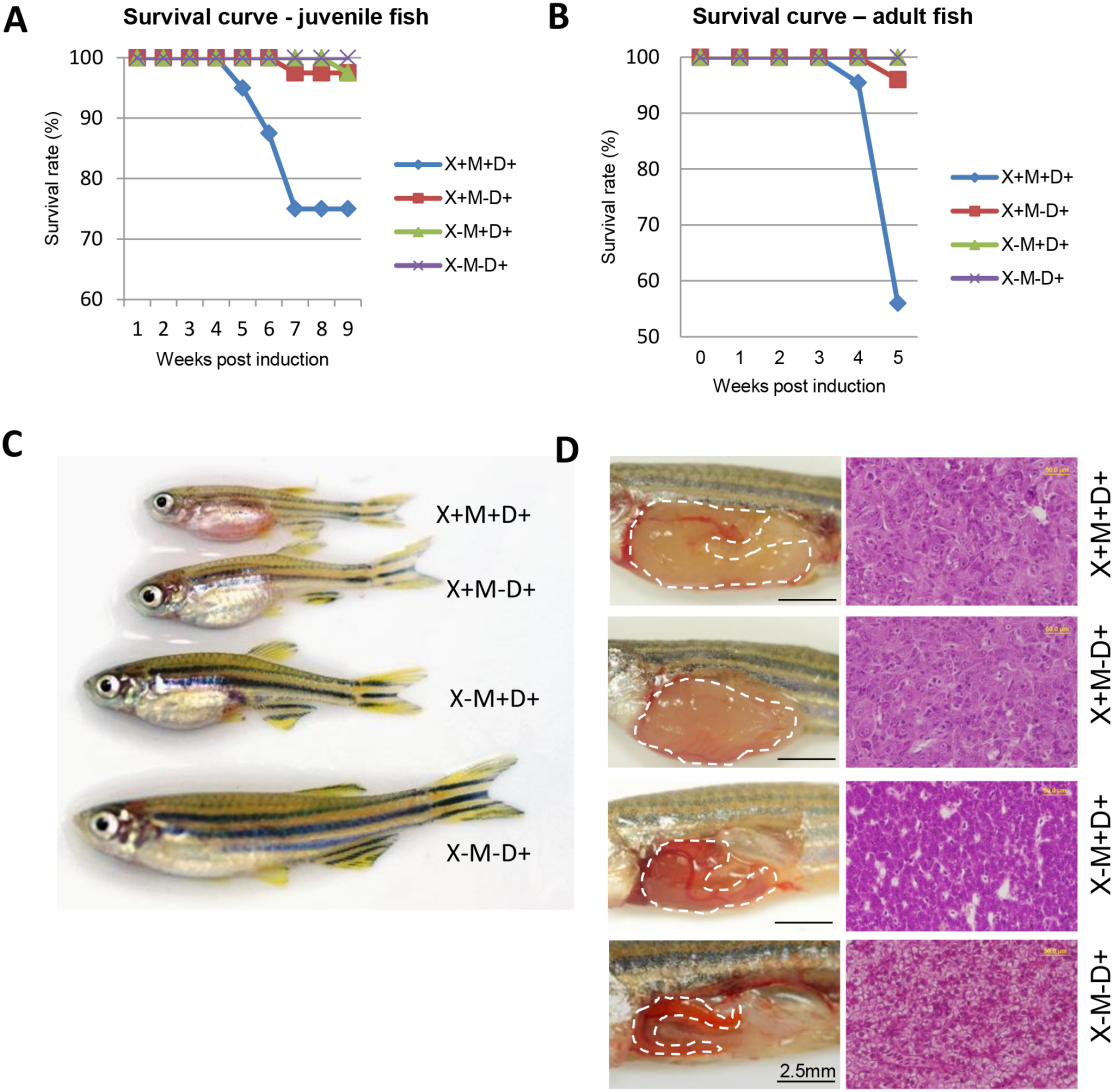
Synergistic effect of *Myc* and *xmrk* oncogenes in transgenic zebrafish survival and liver tumorigenesis. (A,B) Survival curve (A) and gross morphology (B) of oncogene transgenic zebrafish following doxycycline induction at the juvenile stage (starting from 21 dpf). (C) Survival curve of oncogene transgenic zebrafish following doxycycline induction at the adult stage (starting from 3.5 mpf). (D) Gross observation of liver phenotype (left) and histological sections of livers stained by hematoxylin and eosin dyes (right). Abbreviations: X,*xmrk;* M,*Myc;* D, doxycycline treatment.

In order to investigate the synergetic effect of *Myc* and *xmrk* on zebrafish liver tumorigenesis, heterozygous *TO(Myc)* and *TO(xmrk)* were crossed and four groups of fish were resulted: one double-transgenic (M+X+), two single-transgenic (M+X- and M-X+) and one non-transgenic sibling (M-X-). These fish were subjected to doxycycline treatment at juvenile stage (21 dpf). Mortality was first observed in M+X+D+ double-transgenic fish at 4 wpi, whereas no death was observed in the doxycycline-treated single-transgenic fish (M+X-D+ and M-X+D+) and the control groups (M-X-D+) (Fig 1A). The mortality rate of the double transgenic fish quickly reached 25% by 7 wpi, while only small mortalities were observed in the single transgenic fish and no death was found in the controls. At the same time, M+X+D+ fish showed slower growth and obviously enlarged abdomen at 6 wpi (Fig 1B). In comparison, single-transgenic fish showed less severe phenotype and all control fish had normal growth and gross appearance (Fig 1B).

Next, male fish from the same cross were raised to adult and induced with doxycycline from 3.5 mpf. Similarly, the *Myc*/*xmrk* transgenic fish started to show high mortality after 4 wpi (Fig 1C). Previously, high mortality was only observed after 6 weeks of induction in M-X+D+ fish[7]. Obvious enlarged abdomen and overgrowth of liver were observed in the M+X+D+ and M-X+D+ fish, whereas relatively mild liver overgrowth was observed in M+X-D+ fish (Fig 1D). Histopathological diagnosis showed formation of HCC in the *Myc*/*xmrk* and *xmrk* transgenic fish, while only HCA (hepatocellular adenoma) in *My*c fish (Fig 1D, S1 Table). Thus the *Myc*/*xmrk* transgenic fish developed faster and more severe HCC after doxycycline induction than the two other single transgenic lines, indicating a synergetic effect of *Myc* and *xmrk* resulted in carcinogenesis.

### Distinct transcriptomes between tumor and normal livers

**Fig 2:**
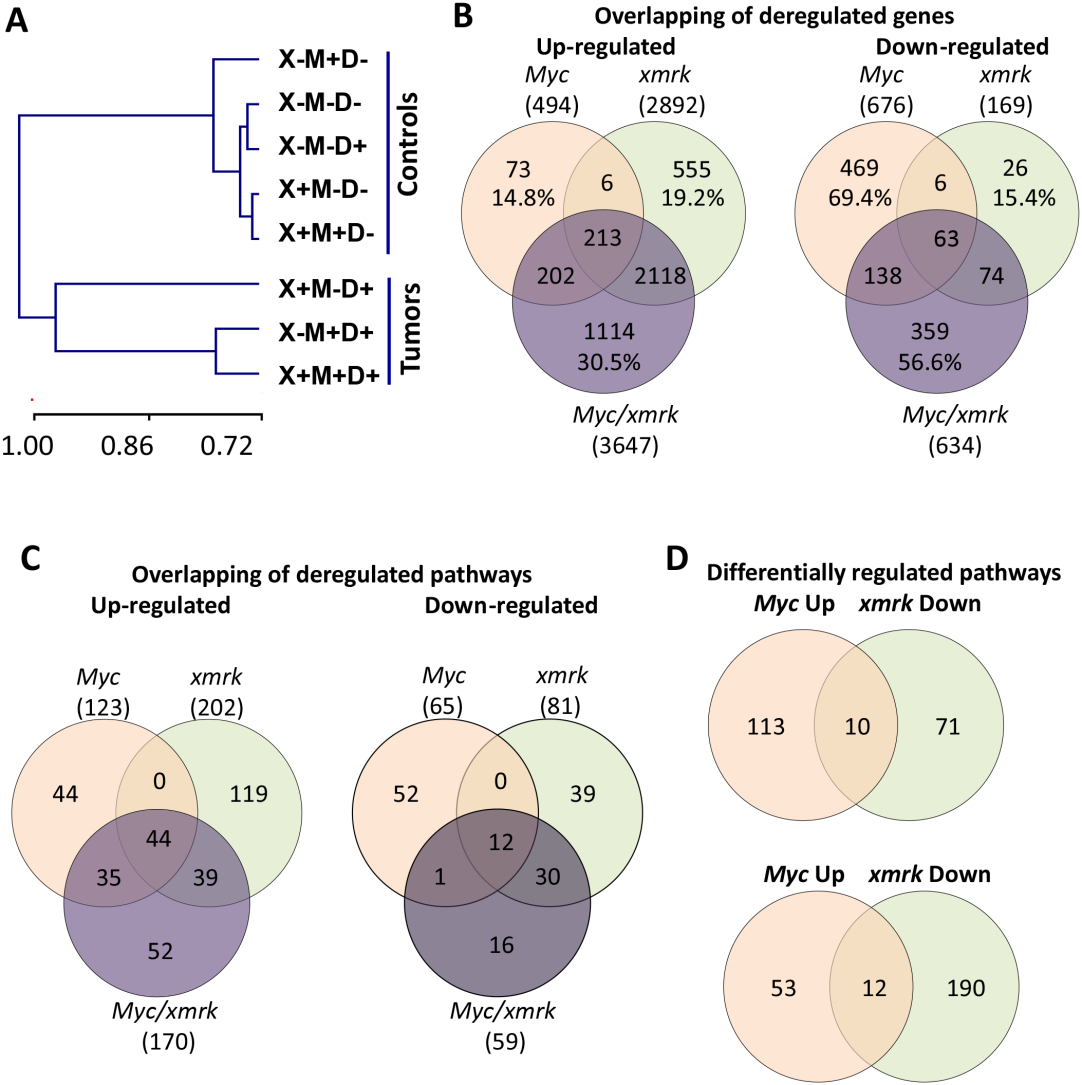
RNA-Seq analyses of *Myc*-, *xmrk*-, *Myc/xmrk*-induced liver tumors. (A) Hierarchical clustering of the eight RNA-Seq samples.(B) Venn diagram of up- and down-regulated transcripts in the three liver tumors. (C) Venn diagram of up-and down-regulated canonical pathways in the three liver tumors. (D) Venn diagram of pathways with opposite directions between *Myc*- and *xmrk*-induced liver tumors.

In order to compare the molecular basis of *Myc* and *xmrk* induced liver tumors and to investigate the molecular mechanism of the synergy of *Myc* and *xmrk*, RNA-Seq was carried out on liver samples from five control groups (M-X-D-, M+X-D-, M-X+D-, M+X+D- and M-X-D+) and three liver tumor groups (M+X+D+, M+X-D+ and M-X+D+). 11–18 million tags were generated from each sample and these tags were mapped to the zebrafish RefSeq mRNA database with mapping efficiency ranged from 22% to 44% (S2 Table). Hierarchical clustering was performed using the entire transcriptome (Fig 2A). Pearson correlation indicated that the five control samples were very similar and they were well separated from the three tumor samples.

### Synergistic effect of *Myc* and *xmrk* oncogenes in double transgenic tumors

To understand the molecular mechanisms of these liver tumors, differentially expressed genes were first identified by comparison of tumor samples with matched control samples. For the *TO(Myc)* and *TO(xmrk)* lines, one tumor and three control samples were compared, i.e. *Myc* tumors (M+X-D+) were compared with three matched controls(M-X-D-, M+X-D- and M-X-D+) and *xmrk* tumors (M-X+D+) with three matched controls(M-X-D-, M-X+D- and M-X-D+). The p value was calculated using one sample t-test. We applied a cutoff at fold change >1.5 and p value < 0.05. To eliminate rare transcripts for functional analyses, we also applied a transcript abundance cutoff at TPM (transcript per million) >10 in either control or tumor samples. With these criteria, there were 494 up-regulated and 676 down-regulated transcripts in the *Myc* tumors (S3 Table), and 2,892 up-regulated and 169 down-regulated transcripts in the xmrk tumors (S4 Table). The double transgenic tumors (M+X+D+) were compared with five control samples (M-X-D-, M+X-D-, M-X+D-, M+X+D- and M-X-D+). With the same statistical method and cutoff, 3,647 and 634 transcripts were found to be up- and down-regulated, respectively (S5 Table).

In order to understand the interaction of *xmrk* and *Myc*, significantly up- and down-regulated genes from two single and one double transgenic tumor samples were compared through the Venn diagram. As shown in Fig 2B, 84.0% of *Myc* up-regulated and 80.6% of *xmrk* up-regulated transcripts were also up-regulated in the *Myc*/*xmrk* transgenic tumors, indicating that the double transgenic tumor maintained most up-regulated transcripts from both single transgenic tumors. Meanwhile, a large portion (30.5%) of up-regulated genes in the double transgenic tumors was unique while the unique portions were only 14.8% and 19.2% for the *Myc* and *xmrk* tumors respectively. In contrast, there were relatively low overlaps in down-regulated genes among the three types of tumors, leaving 69.4% and 56.6% of down-regulated genes unique in *Myc* and double transgenic tumors respectively. There was a relatively small number (169) of down-regulated genes in *xmrk* tumors and the portion of uniquely down-regulated genes was only15.4%.

**Fig 3:**
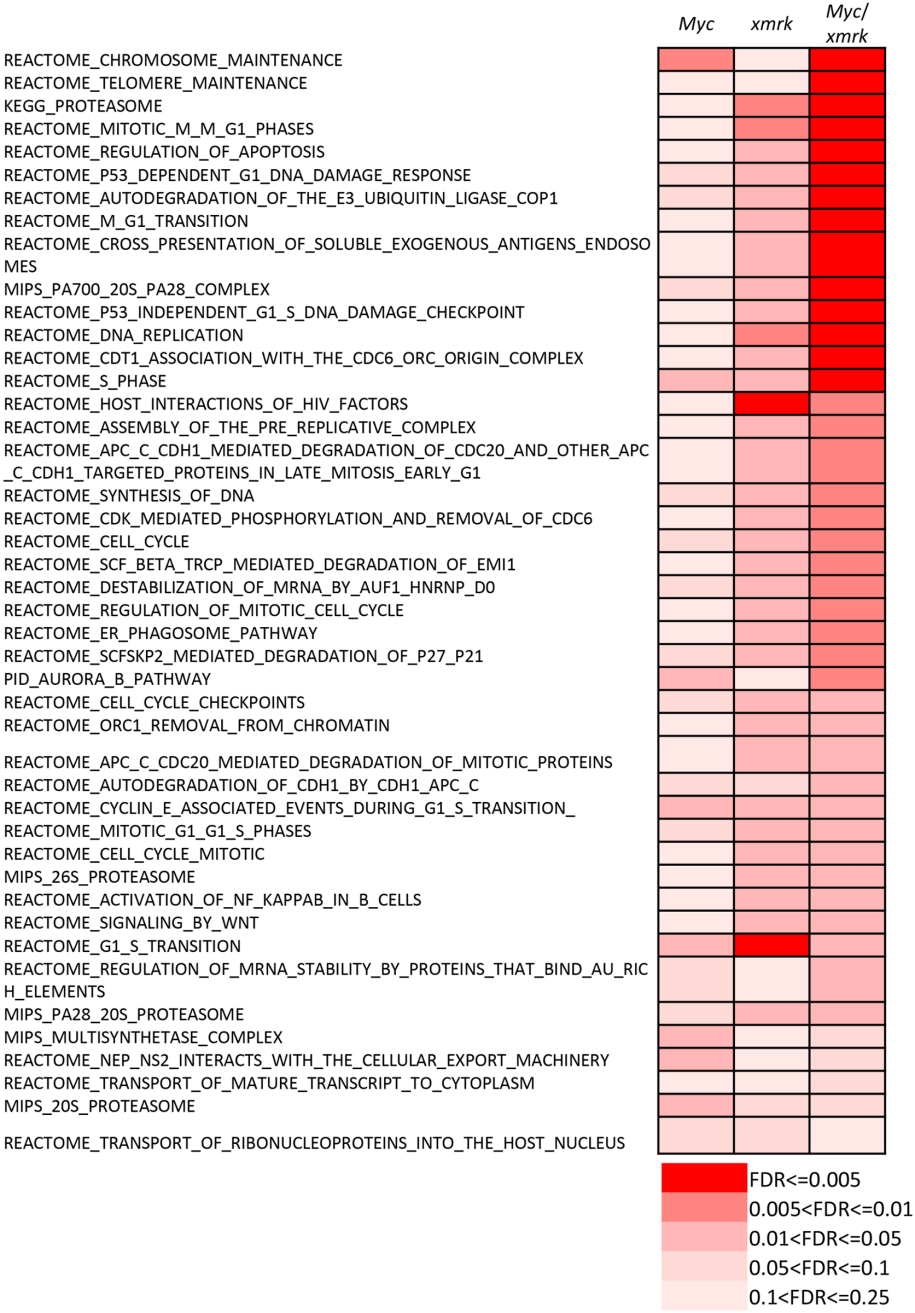
Synergetic effects of pathways co-regulated by *Myc* and *xmrk*. 44 canonical pathways were identified to be up-regulated in both *Myc*- and *xmrk*-induced zebrafish liver cancer. 32 out of them showed more significant up-regulation in the *Myc*/*xmrk* transgenic liver cancer. FDR values are shown in different color gradient as indicated.

Next, biological pathway analysis was performed with these identified differentially expressed genes. Generally, most of the pathways deregulated in the *Myc/xmrk*-induced liver tumors were also deregulated in the *Myc* and/or *xmrk* single transgenic tumors. As shown in the Venn diagram (Fig 2C), all the canonical pathways deregulated (both up and down) in both *Myc*- and *xmrk*-induced zebrafish liver tumors were also deregulated in the *Myc/xmrk* double transgenic liver cancer (S4–S8 Tables). Moreover, the 44 common up-regulated pathways (Fig 2C left) were more significantly up-regulated in the *Myc*/*xmrk* transgenic tumors (Fig 3), including cell cycle regulations, proteasome and telomere maintenance, etc.

**Fig 4:**
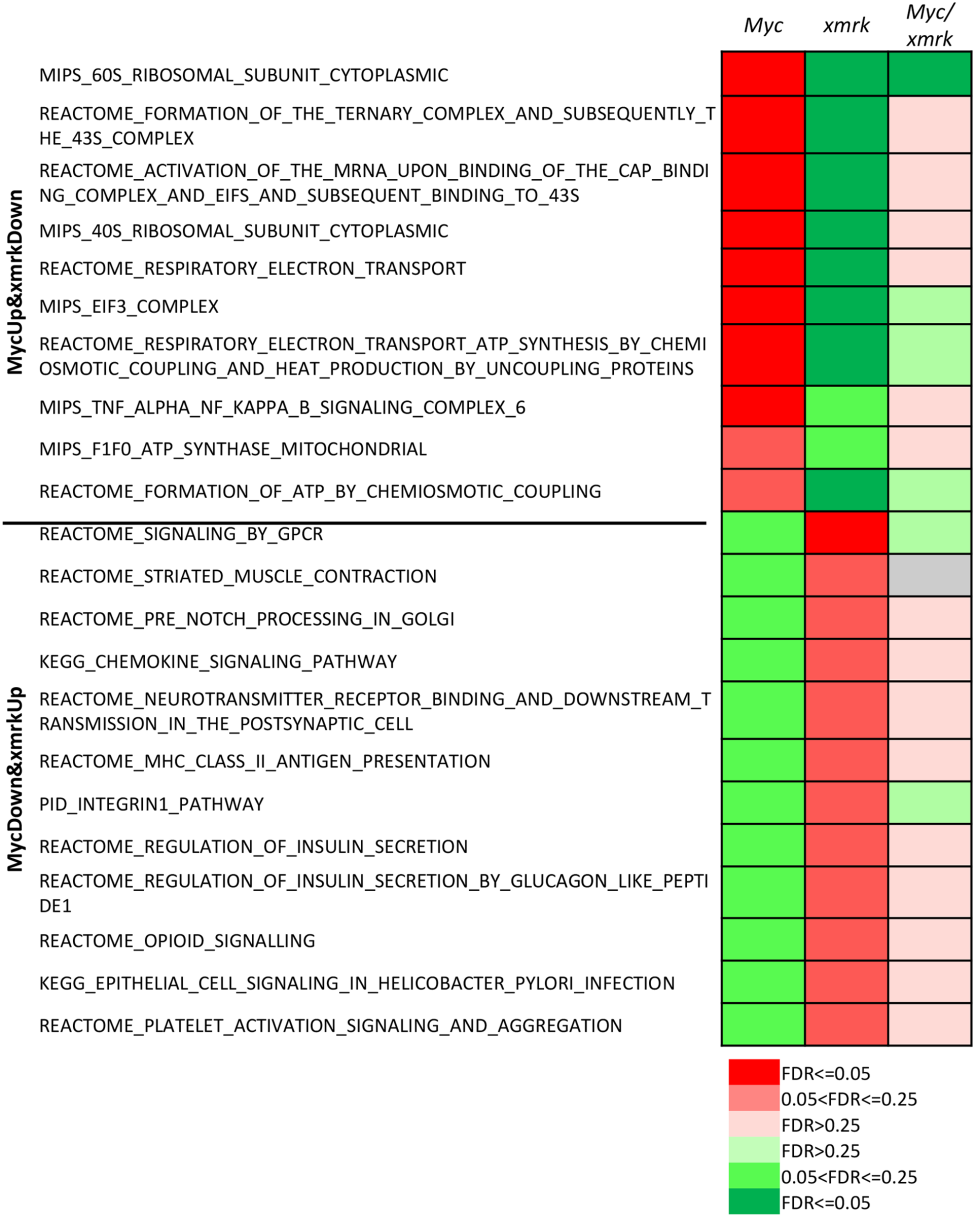
Counteractive effects of pathways oppositely regulated by *Myc* and *xmrk*. All but one pathway which were oppositely regulated by *Myc* and *xmrk* were counterbalanced and did not show any significant changes in the *Myc*/*xmrk* transgenic liver cancer. Red colors indicate up-regulation while green color down-regulation. FDR values are shown in different color gradients as indicated.

Moreover, some pathways showed opposite changes in the *Myc*- and *xmrk*-induced zebrafish liver cancers (Fig 2D). Pathways which were up-regulated by *Myc* but down-regulated by *xmrk* were cytoplasmic ribosome, mitochondrial ATP synthesis and TNFα/NFκB signaling, while pathways which were up-regulated by *xmrk* but down-regulated by *Myc* included GPCR signaling, insulin secretion, chemokine and integrin 1 pathway, and MHC II antigen presentation. These pathways, which were oppositely regulated by *Myc* and *xmrk* (Fig 2D), were counterbalanced in the *Myc*/*xmrk* transgenic liver cancer (Fig 4).

### Molecular changes revealed by uniquely deregulated genes in the *Myc/xmrk* tumors

**Fig 5:**
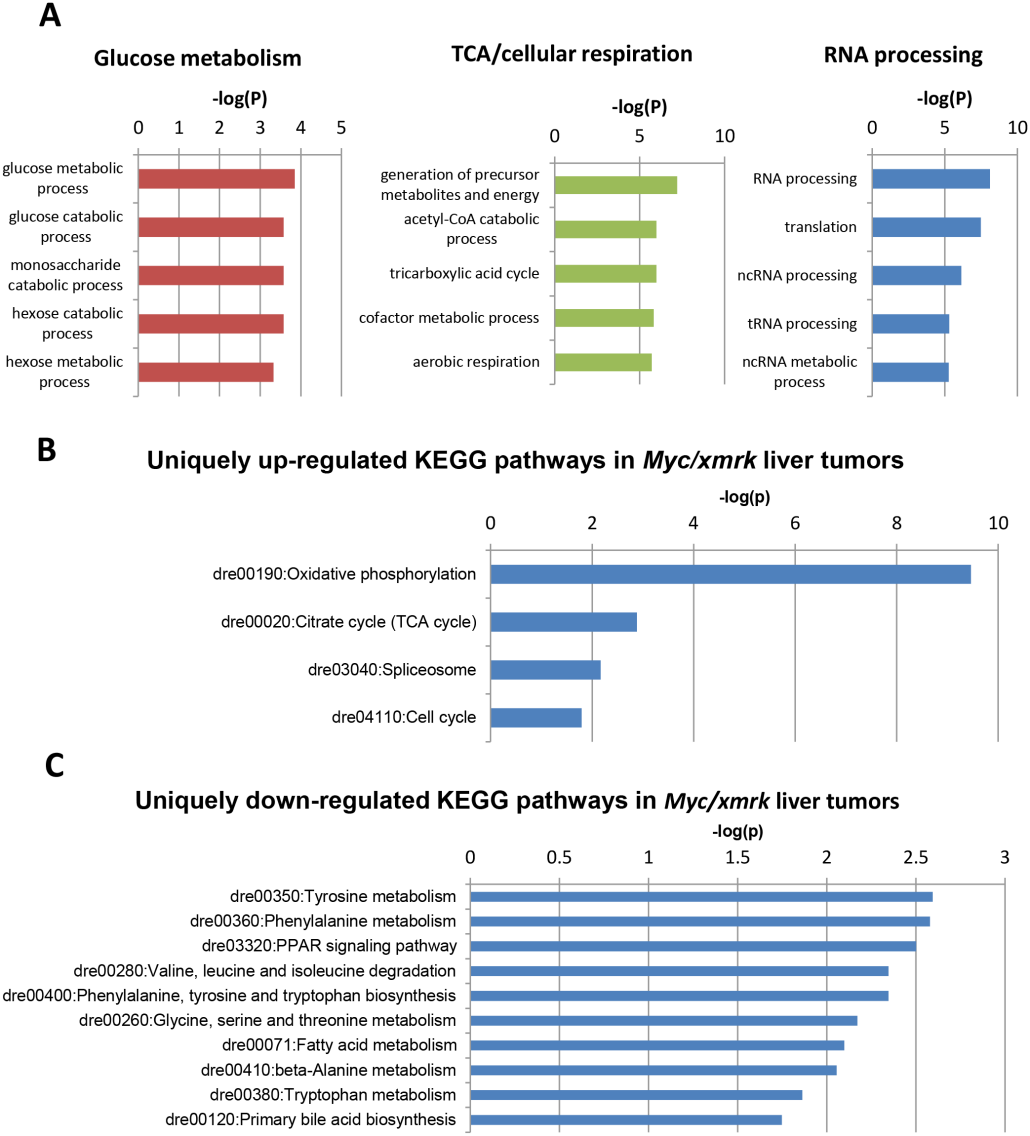
Uniquely up- and down-regulated biological process (BP) and KEGG pathways in *Myc/xmrk* liver tumors. Uniquely up- or down-regulated genes in the *Myc/xmrk* tumors (1,114 and 359 genes respectively as indicated in Fig 2B) were input into the DAVID online software and top BPs and KEGG pathways are shown. (A) Top significantly up-regulated BPs. (B) Significantly up-regulated KEGG pathway with P<0.05 and Benjamini value<0.05 cutoff. (C) Significantly down-regulated KEGG pathway with P<0.05 and Benjamini value<0.05 cutoff. Negative log P-value was plotted against different processes/pathways.

Apart from commonly deregulated genes and pathways, a large portion of deregulated genes (30.5% up and 56.6% down) and biological pathways (30.6% up and 27.1% down) in the *Myc/xmrk* tumors were unique (Fig 2B and 2C).To better understand the biological processes in which these uniquely differentially expressed genes were involved, GO analysis were performed. Both the uniquely up- or down-regulated genes in the *Myc/xmrk* tumors were used as input for DAVID analysis. As shown in Fig 5A, the top up-regulated BP (Biological Process) items were mostly metabolism-related and included three major groups: glucose metabolism, TCA/cellular respiration and RNA processing. KEGG pathway analysis showed similar metabolic pathways analysis such as Oxidative phosphorylation and Citrate cycle (TCA cycle), as well as Spliceosome and Cell cycle, implying that different RNA processing also occurred in the double transgenic tumor (Fig 5B). In contrast, only one GO item, oxidation reduction, was significantly down-regulated in BP items (data not shown), while KEGG analysis showed down-regulation of metabolism of lipid and amino acid, such as PPAR signaling pathway; Fatty acid metabolism; Tyrosine metabolism; and Glycine, serine and threonine metabolism (Fig 5C). Collectively, these analyses suggested significant deregulation of metabolism-related pathways in the *Myc/xmrk* tumors.

### Potential Warburg effect enriched in the *Myc/xmrk* tumors

**Fig 6:**
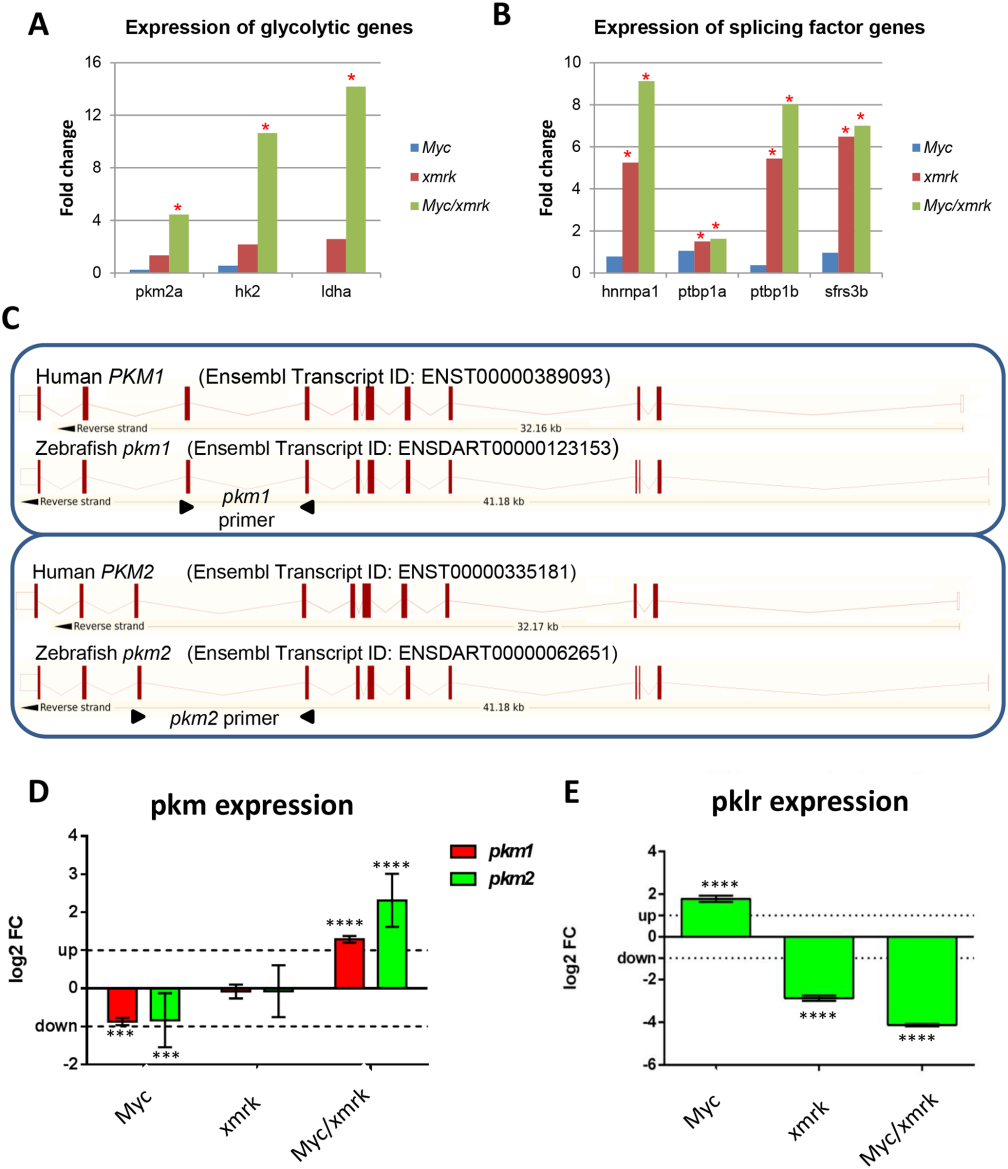
Expression of Warburg effect genes. (A) Expression of glycolytic genes in *Myc*, *xmrk* and *Myc/xmrk* tumors. (B) Expression of glycolytic genes splicing factors in *Myc*, *xmrk* and *Myc/xmrk* tumors. Asterisks indicate significantly changed genes (fold change>1.5, P<0.05). (C) Schematic comparison of genomic structure of human and zebrafish *PKM1/pkm1*and *PKM2/pkm2* isoforms. The primers used for RT-qPCR are indicated by arrowheads. (D) RT-qPCR quantification of zebrafish *pkm1* and *pkm2*expression in three tumor samples as compared with non-tumor controls. ***P<0.001; ****P<0.0001. (E) RT-qPCR quantification of *pklr* expression in three tumor samples as compared with non-tumor controls. ****P<0.0001.

As indicated in Fig 5, several important metabolomic pathways were up-regulated in*Myc/xmrk* tumors. In particular, Acetyl-CoA is an important mediator between glucose, fatty acid and amino acid metabolism and TCA cycle, and these up-regulated metabolic pathways indicated a high requirement of glucose, fatty acid and amino acid metabolism. However, the fatty acid and amino acid metabolism were down-regulated in the *Myc/xmrk* tumors, suggesting a major dependence of energy production through glucose metabolism. Thus, we envisage an existence of Warburg effect in the *Myc/xmrk* tumors, which describes the phenomena that many cancer cells produce energy by a high rate of glycolysis followed by lactic acid fermentation even in the presence of sufficient oxygen [30]. To confirm this, we examined expression of genes for critical enzymes in glycolysis and observed significant up-regulation of glycolytic genes including*hk2*, *pkm* and *ldha* in the *Myc/xmrk* tumors, whereas none of them showed significantly changed in either *Myc* or *xmrk* tumors (Fig 6A), thus strongly suggesting up-regulation of the glycolysis process or Warburg effect uniquely in the *Myc/xmrk* tumors. Moreover, splicing factors for the glycolytic genes including *hnrnpa*, *ptbp1a*, *ptbp1b* and *sfrs3b* were all further up-regulated in the *Myc/xmrk* tumors compared with the *xmrk* tumor, whereas no significant change was observed in the*Myc*tumors (Fig 6B), indicating potentially different splicing activities in these different types of tumors.

It is well known that two major transcript isoforms of *PKM* exist in human: *PKM1*, expressed in adult tissues;*PKM2*, expressed in embryonic and adult tissues and also enriched in many cancers [31]. To determine whether the two isoforms similarly exist in zebrafish, genomic sequence of zebrafish *pkm*, the human *PKM* orthologue gene, was examined. As illustrated in Fig 6C, zebrafish *pkm* has two isoforms, *pkm202*and *pkm201*, each of which has high conservation in gene structure with human *PKM1* and *PKM2* isoforms respectively. Similar to human *PKM1* and *PKM2*, which have mutually exclusive exons 9 and 10 [32], Zebrafish *pkm202* and *pkm201*also have mutually exclusive exons 10 and 11. Thus, these two isoforms were designated *pkm1* and *pkm2* in accordance with human gene nomenclature in the following description.

To determine if these zebrafish isoforms were up-regulated in tumors, isoform specific primers were designed against exons 9, 10 and 11 in order to distinguish the two isoforms (Fig 6C). RT-qPCR was performed with these primers and significant up-regulation of *pkm2* and *pkm1* (to a less extent) in the *Myc/xmrk* tumors was found in comparison with control samples. In contrast, the *xmrk* tumors showed no significant changes in both isoforms, whereas the *Myc* tumors showed significant down-regulation of both isoforms. Thus, there is a unique enrichment of *pkm2* and *pkm1* isoforms in the *Myc/xmrk* tumors. There are two pyruvate kinase genes, *PKM/pkm* and *PKLR/pklr* in both human and zebrafish genomes. We noted that zebrafish *pklr* had opposite expression changes, significant up-regulation in *Myc* tumors and down-regulation in *xmrk* and *Myc/xmrk* tumors (Fig 6E). Thus, different types of tumors had differential regulation of pyruvate kinases and subsequent metabolism.

We also measured the expression of *hk2*, *ldha*, *pkm1* and *pkm2* in both pre-tumors (one week after Dox induction) and HCC (three week after induction) from adult *Myc/xmrk* transgenic zebrafish and we found that *hk2* and *pkm2* showed up-regulation even from the pre-tumor stage while *ldha* and *pkm1* were up-regulated only in the HCC stage (S1 Fig).These molecular data indicate an enhanced Warburg effect in the HCC stage.

### Suppression of oncogenic growth of liver by Pkm2 activator

**Fig 7:**
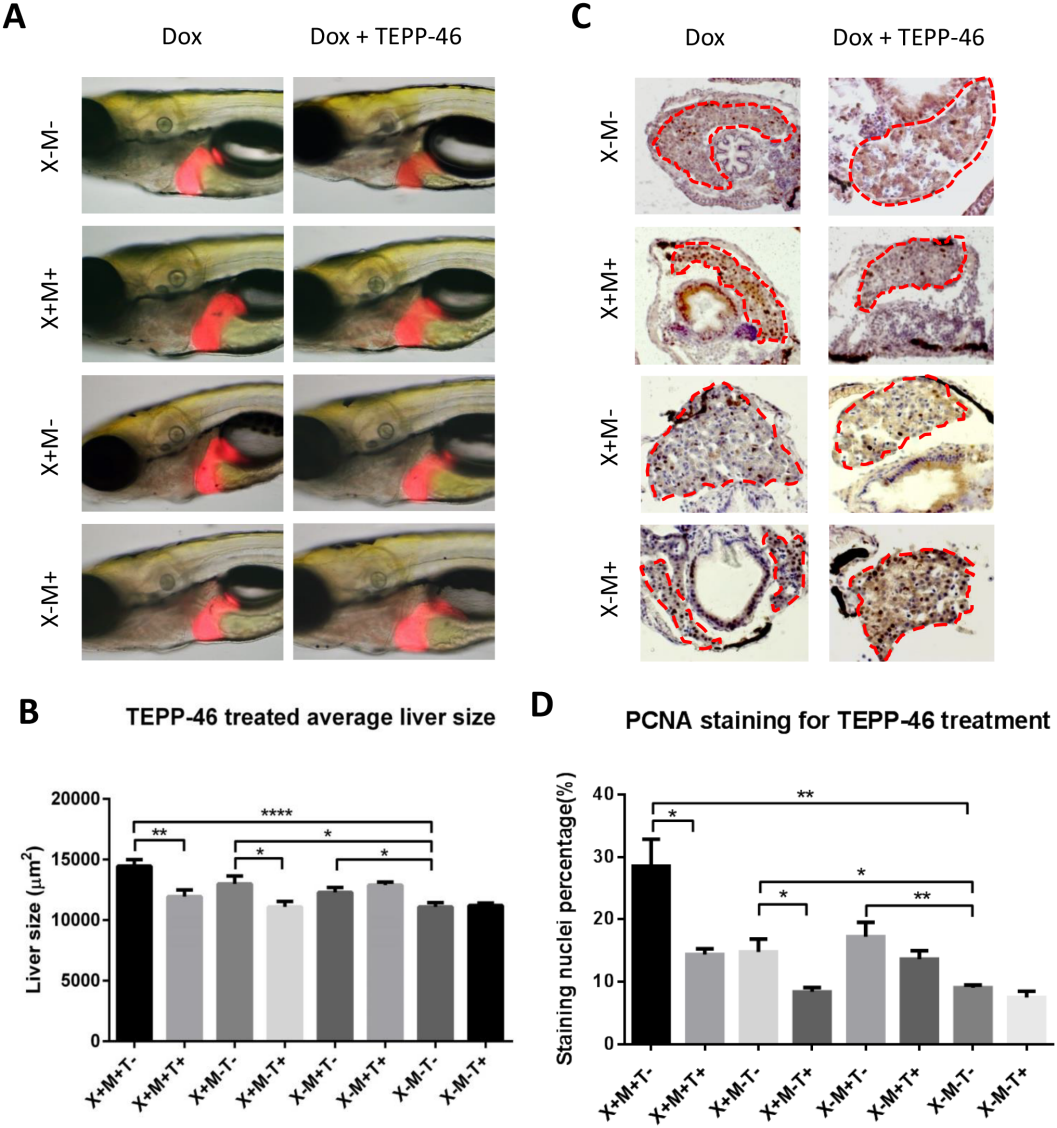
Suppression of growth of oncogenic livers in *Myc/xmrk* double transgenic larvae by Pkm2 activation. 10 μg/ml TEPP-46 was used to treat zebrafish larvae from 4 dpf for 96 hours and 2D liver size was measured by using ImageJ (1.49J) and cell proliferation on cryosections were examined by immune-staining of PCNA. (A,B) Changes of 2D liver size. Representative images from each group are shown in (A) and quantification of 2D liver size is presented in (B). (C,D) Immunostaining of PCNA for cell proliferation. Representative images in the liver area from each experimental group are shown in (A) and quantification of PCNA positive cells as percentage of liver cells is presented in (B). Group designations: X for *xmrk*, M for *Myc*, and T for TEPP-46. + and—indicate presence and absence respectively. All groups also received 30 μg/ml doxycycline in the experimental duration (4–8 dpf). Statistical significance was examined by unpaired student t test. *P<0.05; **P<0.01; ****P<0.0001.

It has been reported that PKM2 has two forms: an active tetrameric form and a less active dimeric form; tumor cells have predominantly the inactive dimeric form, which causes incomplete glycolysis and anabolism to promote tumor growth [33, 34]. Thus, increase of pyruvate kinase activity results in inhibition of tumor growth; this has been demonstrated by using PKM2 specific activator, TEPP-46, to stabilize the tetrameric configuration of PKM2 [33]. To demonstrate a similar mechanism in the zebrafish liver cancer model, TEPP-46 was used to treat all the three transgenic larvae together with doxycycline. As shown in Fig 7A and 7B, all three transgenic larvae showed significantly enlarged livers when doxycycline was administrated at 30 μg/ml, with the *Myc/xmrk* larvae showing the highest enlargement. When TEPP-46 was applied together with doxycycline, *Myc/xmrk* larvae showed the most significant reduction in liver size (P = 0.0026) compared with milder reduction in *xmrk* larvae (P = 0.024) and no significant change in *Myc* larvae. To further understand the mechanism of TEPP-46 on liver reduction, PCNA staining were performed. As shown in Fig 7C and 7D, significant down-regulation of cell proliferation in the liver occurred in the TEPP-46 treated *Myc/xmrk* and *xmrk* larvae, and again there was no significant effect on *Myc* larvae. These observations suggested that Pkm2 enzyme activity is indeed involved in liver carcinogenesis through regulating cell proliferation.

## Discussion

Warburg effect was first described by Otto Warburg ninety years ago based on an observation in rat liver carcinoma that does not consume more oxygen than the normal liver tissue and produces lactic acid even in the presence of oxygen [30]. Later, this phenomenon was proven as a common metabolic characteristic shared by many human cancers, where there are increased uptake of glucose and conversion of glucose into lactate with a high rate of glycolysis even in the presence of adequate oxygen, unlike normal cells that use only a low rate of glycolysis followed by oxidation of pyruvate in mitochondria [35]. Hepatocellular carcinoma (HCC), the major malignant tumor of liver has been shown to have moderate Warburg effect compared to many other types of cancers [36, 37]. Currently, only three HCC metabolomic profiles have been reported from human samples and they all showed a consistent shift to glycolysis in HCC samples [37–39]. However, the intracellular signaling which triggers the glycolytic changes in HCC is still unknown.

In the present study, we found through transcriptomic analyses that there was a potential Warburg effect in zebrafish HCC with synergistic effect of EGFR and Myc pathways. Warburg effect has often been misinterpreted as increased glycolysis at the expense of damaged mitochondrial respiration. However, Koppenol et al. have reinforced the correct interpretation of Warburg effect in their recent review that both aerobic glycolysis and respiration occurred concurrently in the cancer cells [35]. Consistent with this notion, we observed elevated glycolysis as well as Citrate cycle (TCA cycle)/Oxidative phosphorylation in our zebrafish *Myc/xmrk* HCC model, further confirming the Warburg effect.

Myc has long been linked to Warburg effect due to its ability to regulate expression of many glycolytic genes, including glucose metabolism genes, such as *GLUT1* (glucose transporter), *HK2* (hexokinase 2), *PKM2* (pyruvate kinase isozymes M2), *LDHA* (lactate dehydrogenase A) [40, 41]. However, in our current data, no obvious changes or even down-regulation were observed in the critical glycolytic genes such as *hk2*, *ldha* and *pkm2* in the *Myc* tumors, although other Myc target genes such as ribosome and mitochondria genes were obviously up-regulated. These data could be due to the tumor stage as we only investigated 6 weeks after induction and we obtained only hepatocellular adenoma at this stage (S1 Table). Alternatively, our observation may reveal a different dependence of Myc pathway in metabolism changes in liver tumor. Similar to this notion, Nilsson et al. have recently shown that cell-context dependence of Myc-regulated metabolism changes in cancer formation in B-cell lymphoma in a transgenic mouse model. By crossing the B-cell lymphorma transgenic line with *Ldha* knockout mouse, they have found that *Ldha*is dispensable for lymphoma formation driven by Myc [42]. However, *Ldha* was indispensable for fibroblast tumor formation driven by Ras [42]. Thus valid *in vivo* models for further metabolism analyses under different signaling pathways will be highly desired.

EGFR has been linked to Warburg effect only recently and all experiments were based on *in vitro* cancer cell lines. EGFR activates ERK1/2-dependant nuclear translocation of PKM2 and activates *Myc*, which in turn up-regulate *GLUT1*, *LDHA* and *PKM2* expression in human glioblastoma multiforme (GBM) cells [41]. It has also been shown that the non-metabolism function of EGFR activates PKM2 in β-catenin transactivation and subsequent cell proliferation and tumorigenesis in GBM, prostate and breast cancer cell lines [43, 44]. Different from these observations, in our zebrafish models, EGFR alone did not trigger significant up-regulation of Warburg effect, although HCC was observed in 100% induced *TO(xmrk)*fish [7]. Thus, our observations indicated a liver tumor specific requirement of EGFR and Myc pathway in regulation of Warburg effect in hepatocarcinogenesis.

Compared to either Myc or EGFR (Xmrk) single pathway activated liver tumors, *Myc/xmrk* tumors have a major shift of metabolism process towards to a Warburg effect. This is in sharp contrast to either no change or down-regulation in glycolytic gene expression in *xmrk* or *Myc* alone tumors. Among these critical glycolytic genes, *PKM* is the best documented gene for Warburg effect and different splicing isoforms have been linked to many different tumors. Although early reports have indicated that the *PKM2* isoform is specifically expressed in the tumor, a recent study from a survey of 16 human normal adult tissues and cancer samples has demonstrated that both PKM1 and PKM2 isoforms exist in normal tissues while PKM2 is the dominant isoform in both normal and cancers in majority of tissues, albeit with a more obvious enrichment of PKM2 in the cancer samples [31]. PKM expression in the normal liver is rather low in all the normal tissues studied and the extent of PKM2enrichment between tumor and control is higher in HCC than in majority of cancers from other tissues [31]. Consistent to this notion, we observed significant enrichment of both *pkm2*and *pkm1*isoforms in our *Myc/xmrk* tumor samples, with a more obvious enrichment of *pkm2*isoform.Currently, the information on function of PKM1 and PKM2 in different cancers is controversial, as some studies indicated indispensable function of PKM2 in glioblastoma formation [41, 43] while others showed dispensable function of PKM2 in colon cancer maintenance and progression [45]. This could be due to different experimental systems, such as cell line versus xenograft models, different cancer type, different cancer drivers, etc. Here, our *Myc/xmrk* transgenic zebrafish provide a context-specific, tumor-specific and pathway-specific *in vivo* model to address these questions. For example, in the Pkm2 activator experiment, significant suppression of oncogenic liver growth in both *Myc/xmrk* and *xmrk*fish was observed, in contrast to the lack of significant change in the *Myc*fish. This phenomenon was further demonstrated to be due to cell proliferation regulation as both *Myc/xmrk* and *xmrk* fish showed significant reduction in cell proliferation, whereas *Myc* fish did not show the reduction. These data suggested a potential function of Xmrk (EGFR) on cell proliferation through regulating Pkm2 enzyme activity.

In summary, we have established an *in vivo* HCC model with obvious Warburg effect through synergistic interaction of EGFR and Myc pathways. The *Myc/xmrk* transgenic zebrafish reported here should be valuable for further investigation of molecular mechanism of Warburg effect in HCC *in vivo* and for chemical screening in development of anti-metabolism therapies for HCC. In our previous comparative transcriptomic analyses with the zebrafish and human HCC data, we found that about 17% human HCCs share molecular signatures of over-expression of *MYC* and *EGFR* oncogenes [26]. By examining the HCC data from the TCGA database (http://cancergenome.nih.gov), we also found that 19.6% of human HCC samples show up-regulation (>1.5 fold) of both *EGFR* and *MYC* (data not shown). Thus, these subsets of human HCCs may have Warburg effect and further effort should be directed to investigation of our findings in human HCC samples in terms of the relationship of signaling pathway and HCC metabolism, which should benefit diagnosis and prognosis of human patients.

## Supporting Information

S1 Fig Expression of Warburg effect genes in pre-tumors and HCC of *Myc/xmrk* transgenic zebrafish. *Myc/xmrk* transgenic zebrafish were induced by doxycycline for 1 week (pre-tumor) and 3 weeks (HCC) and livers were collected for RNA extraction and RT-qPCR. Expression values in pre-tumor/tumor samples are compared with those in non-tumor controls. ****P<0.0001.
(PDF)

S1 Table Summary of histopathological examination of liver tumor formation in transgenic zebrafish. (DOCX)

S2 Table Summary of RNA-Seq data. (DOCX)

S3 Table Up- and down-regulated genes in *Myc*-induced liver tumors. (XLSX)

S4 Table Up- and down-regulated genes in *xmrk*-induced liver tumors. (XLSX)

S5 Table Up- and down-regulated genes in *Myc*/*xmrk-*induced liver tumors. (XLSX)

S6 Table Differentially expressed canonical pathways in *Myc*-induced liver tumors. (DOCX)

S7 Table Differentially expressed canonical pathways in *xmrk*-induced liver tumors. (DOCX)

S8 Table Differentially expressed canonical pathways in *Myc*/*xmrk-*induced liver tumors. (DOCX)

S1 Text The ARRIVE Guidelines Checklist. (PDF)
