# Supplementary material for: Synergistic Induction of Potential Warburg Effect in Zebrafish Hepatocellular Carcinoma by Co-Transgenic Expression of Myc and xmrk Oncogenes
Source: PLoS One. 2015 Jul 6;10(7):e0132319. doi: 10.1371/journal.pone.0132319 (PMC4492623; doi:10.1371/journal.pone.0132319)
Supplement: S1 Fig — Myc/xmrk transgenic zebrafish were induced by doxycycline for 1 week (pre-tumor) and 3 weeks (HCC) and livers were collected for RNA extraction and RT-qPCR. Expression values in pre-tumor/tumor samples are compared with those in non-tumor controls. ****P<0.0001. (PDF) [file pone.0132319.s001.pdf]

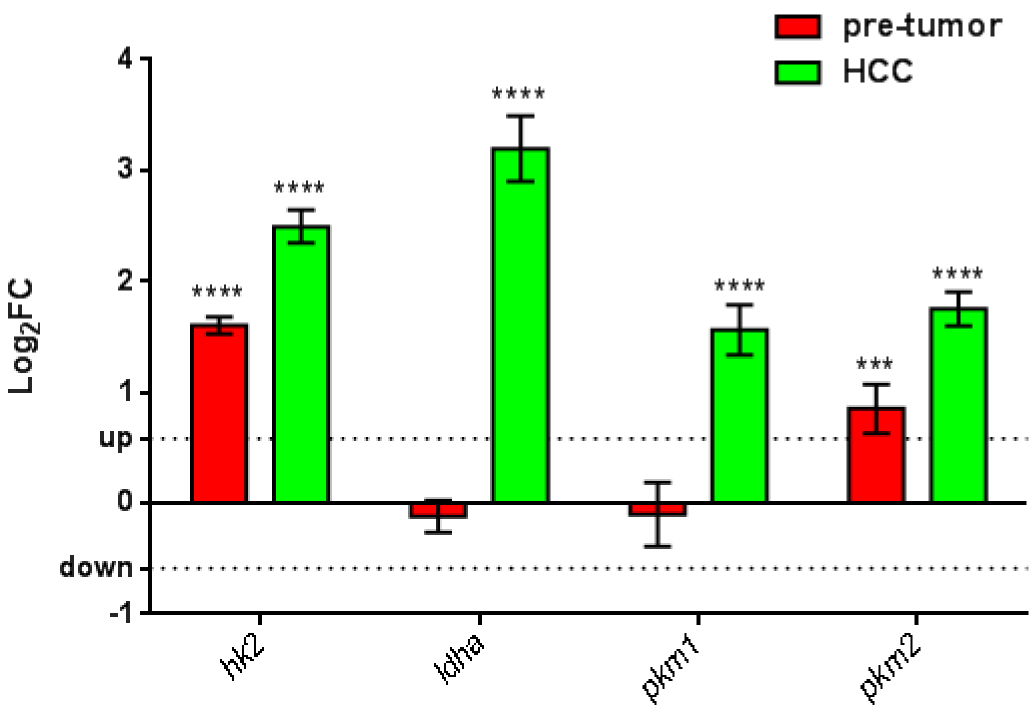

S1 Fig. Expression of Warburg effect genes in pretumors and HCC of *Myc/xmrk* transgenic zebrafish. *Myc/xmrk* transgenic zebrafish were induced by doxycycline for 1 week (pre-tumor) and 3 weeks (HCC) and livers were collected for RNA extraction and RT-qPCR. Expression values in pre-tumor/tumor samples are compared with those in non-tumor controls. ****P<0.0001.
