# Supplementary material for: Synergistic Induction of Potential Warburg Effect in Zebrafish Hepatocellular Carcinoma by Co-Transgenic Expression of Myc and xmrk Oncogenes
Source: PLoS One. 2015 Jul 6;10(7):e0132319. doi: 10.1371/journal.pone.0132319 (PMC4492623; doi:10.1371/journal.pone.0132319)
Supplement: S1 Table — (DOCX) [file pone.0132319.s002.docx]

**S1 Table. Summary of histopathological examination of liver tumor formation in transgenic zebrafish**

| Sampling time points | Groups | Number | Histopathological features | | | |
| --- | --- | --- | --- | --- | --- | --- |
|  |  |  | HCC | HCA | HCA/HP | Normal |
| 4 wpi | X+M+D+ | 4 | 4 (100%) | 0 | 0 | 0 |
|  | X+M+D- | 5 | 0 | 0 | 0 | 5 |
| 6 wpi | X+M-D+ | 5 | 5 (100%) | 0 | 0 | 0 |
|  | X+M-D- | 5 | 0 | 0 | 0 | 5 (100%) |
| 6 wpi | X-M+D+ | 5 | 0 | 3 (60%) | 2 (40%) | 0 |
|  | X-M+D- | 5 | 0 | 0 | 0 | 5 (100%) |
| 6 wpi | X-M-D+ | 5 | 0 | 0 | 0 | 5 (100%) |
|  | X-M-D- | 5 | 0 | 0 | 0 | 5 (100%) |

HCC, hepatocellular carcinoma; HCA, hepatocellular adenoma; HP, hyperplasia.
