# Supplementary material for: Synergistic Induction of Potential Warburg Effect in Zebrafish Hepatocellular Carcinoma by Co-Transgenic Expression of Myc and xmrk Oncogenes
Source: PLoS One. 2015 Jul 6;10(7):e0132319. doi: 10.1371/journal.pone.0132319 (PMC4492623; doi:10.1371/journal.pone.0132319)
Supplement: S2 Table — (DOCX) [file pone.0132319.s003.docx]

**S2 Table. Summary of RNA-seq data**

|  | Total tag reads | Total uniquely mapped tag reads allowing maximum 2 mismatches | Mapping efficiency | RefSeq entries |
| --- | --- | --- | --- | --- |
| X-M-D- | 14,937,923 | 6,233,555 | 41.73% | 13,049 |
| X-M+D- | 11,929,959 | 4,472,595 | 37.49% | 12,701 |
| X+M-D- | 18,129,256 | 3,939,173 | 21.73% | 11,663 |
| X+M+D- | 14,642,757 | 3,698,713 | 25.26% | 11,137 |
| X-M-D+ | 14,390,779 | 5,021,812 | 34.89% | 12,582 |
| X-M+D+ | 14,998,311 | 5,152,465 | 34.35% | 11,666 |
| X+M-D+ | 10,500,931 | 3,214,091 | 31.61% | 12,414 |
| X+M+D+ | 14,284,481 | 4,736,554 | 33.16% | 14,009 |
