# Supplementary material for: Synergistic Induction of Potential Warburg Effect in Zebrafish Hepatocellular Carcinoma by Co-Transgenic Expression of Myc and xmrk Oncogenes
Source: PLoS One. 2015 Jul 6;10(7):e0132319. doi: 10.1371/journal.pone.0132319 (PMC4492623; doi:10.1371/journal.pone.0132319)
Supplement: S6 Table — (DOCX) [file pone.0132319.s007.docx]

**S6 Table. Differentially expressed canonical pathways in the *Myc*-induced zebrafish liver tumors**

**Up-regulated canonical pathways in the *Myc*-induced zebrafish liver cancer**

| NAME | SIZE | NES | p-val | FDR |
| --- | --- | --- | --- | --- |
| MIPS_RIBOSOME_CYTOPLASMIC | 65 | 3.98 | 0.00E+00 | 0.00E+00 |
| REACTOME_3_UTR_MEDIATED_TRANSLATIONAL_REGULATION | 78 | 3.96 | 0.00E+00 | 0.00E+00 |
| REACTOME_PEPTIDE_CHAIN_ELONGATION | 66 | 3.96 | 0.00E+00 | 0.00E+00 |
| REACTOME_INFLUENZA_VIRAL_RNA_TRANSCRIPTION_AND_REPLICATION | 67 | 3.95 | 0.00E+00 | 0.00E+00 |
| REACTOME_INFLUENZA_LIFE_CYCLE | 80 | 3.95 | 0.00E+00 | 0.00E+00 |
| REACTOME_TRANSLATION | 103 | 3.95 | 0.00E+00 | 0.00E+00 |
| KEGG_RIBOSOME | 67 | 3.94 | 0.00E+00 | 0.00E+00 |
| REACTOME_SRP_DEPENDENT_COTRANSLATIONAL_PROTEIN_TARGETING_TO_MEMBRANE | 81 | 3.88 | 0.00E+00 | 0.00E+00 |
| MIPS_NOP56P_ASSOCIATED_PRE_RRNA_COMPLEX | 72 | 3.86 | 0.00E+00 | 0.00E+00 |
| REACTOME_NONSENSE_MEDIATED_DECAY_ENHANCED_BY_THE_EXON_JUNCTION_COMPLEX | 72 | 3.81 | 0.00E+00 | 0.00E+00 |
| REACTOME_METABOLISM_OF_PROTEINS | 173 | 3.60 | 0.00E+00 | 0.00E+00 |
| REACTOME_METABOLISM_OF_MRNA | 115 | 3.55 | 0.00E+00 | 0.00E+00 |
| REACTOME_METABOLISM_OF_RNA | 130 | 3.51 | 0.00E+00 | 0.00E+00 |
| MIPS_60S_RIBOSOMAL_SUBUNIT_CYTOPLASMIC | 39 | 3.46 | 0.00E+00 | 0.00E+00 |
| REACTOME_FORMATION_OF_THE_TERNARY_COMPLEX_AND_SUBSEQUENTLY_THE_43S_COMPLEX | 36 | 3.34 | 0.00E+00 | 0.00E+00 |
| REACTOME_ACTIVATION_OF_THE_MRNA_UPON_BINDING_OF_THE_CAP_BINDING_COMPLEX_AND_EIFS_AND_SUBSEQUENT_BINDING_TO_43S | 40 | 3.28 | 0.00E+00 | 0.00E+00 |
| MIPS_40S_RIBOSOMAL_SUBUNIT_CYTOPLASMIC | 26 | 3.16 | 0.00E+00 | 0.00E+00 |
| MIPS_TRBP_CONTAINING_COMPLEX_1 | 21 | 2.93 | 0.00E+00 | 0.00E+00 |
| MIPS_55S_RIBOSOME_MITOCHONDRIAL | 33 | 2.54 | 0.00E+00 | 0.00E+00 |
| REACTOME_CYTOSOLIC_TRNA_AMINOACYLATION | 13 | 2.46 | 0.00E+00 | 4.04E-05 |
| MIPS_39S_RIBOSOMAL_SUBUNIT_MITOCHONDRIAL | 22 | 2.50 | 0.00E+00 | 4.25E-05 |
| REACTOME_TRNA_AMINOACYLATION | 13 | 2.37 | 0.00E+00 | 7.61E-05 |
| KEGG_AMINOACYL_TRNA_BIOSYNTHESIS | 11 | 2.28 | 0.00E+00 | 1.79E-04 |
| REACTOME_FORMATION_OF_TUBULIN_FOLDING_INTERMEDIATES_BY_CCT_TRIC | 8 | 2.24 | 0.00E+00 | 3.49E-04 |
| REACTOME_PREFOLDIN_MEDIATED_TRANSFER_OF_SUBSTRATE_TO_CCT_TRIC | 12 | 2.21 | 0.00E+00 | 3.69E-04 |
| KEGG_PARKINSONS_DISEASE | 55 | 2.13 | 0.00E+00 | 1.50E-03 |
| REACTOME_ASSOCIATION_OF_TRIC_CCT_WITH_TARGET_PROTEINS_DURING_BIOSYNTHESIS | 10 | 2.13 | 0.00E+00 | 1.56E-03 |
| REACTOME_CHROMOSOME_MAINTENANCE* | 9 | 2.02 | 2.02E-03 | 6.82E-03 |
| REACTOME_METABOLISM_OF_NON_CODING_RNA | 16 | 2.03 | 2.11E-03 | 6.83E-03 |
| REACTOME_RESPIRATORY_ELECTRON_TRANSPORT | 42 | 2.00 | 0.00E+00 | 8.93E-03 |
| MIPS_EIF3_COMPLEX | 7 | 1.96 | 0.00E+00 | 1.31E-02 |
| REACTOME_RESPIRATORY_ELECTRON_TRANSPORT_ATP_SYNTHESIS_BY_CHEMIOSMOTIC_COUPLING_AND_HEAT_PRODUCTION_BY_UNCOUPLING_PROTEINS_ | 50 | 1.95 | 0.00E+00 | 1.39E-02 |
| MIPS_TNF_ALPHA_NF_KAPPA_B_SIGNALING_COMPLEX_6 | 9 | 1.95 | 0.00E+00 | 1.42E-02 |
| REACTOME_TCA_CYCLE_AND_RESPIRATORY_ELECTRON_TRANSPORT | 69 | 1.94 | 0.00E+00 | 1.58E-02 |
| MIPS_MULTISYNTHETASE_COMPLEX* | 5 | 1.92 | 0.00E+00 | 1.80E-02 |
| REACTOME_PROTEIN_FOLDING | 16 | 1.92 | 0.00E+00 | 1.82E-02 |
| REACTOME_MRNA_PROCESSING | 41 | 1.91 | 1.99E-03 | 1.94E-02 |
| REACTOME_PURINE_RIBONUCLEOSIDE_MONOPHOSPHATE_BIOSYNTHESIS | 7 | 1.91 | 0.00E+00 | 1.97E-02 |
| MIPS_20S_PROTEASOME* | 7 | 1.90 | 4.34E-03 | 1.99E-02 |
| BIOCARTA_EIF2_PATHWAY | 5 | 1.89 | 0.00E+00 | 2.22E-02 |
| PID_MYC_ACTIVPATHWAY | 26 | 1.86 | 0.00E+00 | 2.99E-02 |
| KEGG_HUNTINGTONS_DISEASE | 65 | 1.84 | 0.00E+00 | 3.60E-02 |
| REACTOME_S_PHASE* | 28 | 1.82 | 2.12E-03 | 3.96E-02 |
| REACTOME_PROCESSING_OF_CAPPED_INTRON_CONTAINING_PRE_MRNA | 37 | 1.82 | 3.94E-03 | 4.00E-02 |
| REACTOME_NEP_NS2_INTERACTS_WITH_THE_CELLULAR_EXPORT_MACHINERY* | 10 | 1.82 | 1.05E-02 | 4.02E-02 |
| MIPS_SMN_COMPLEX | 5 | 1.80 | 4.18E-03 | 4.53E-02 |
| REACTOME_G1_S_TRANSITION* | 29 | 1.80 | 4.26E-03 | 4.54E-02 |
| PID_AURORA_B_PATHWAY* | 6 | 1.79 | 6.29E-03 | 4.76E-02 |
| REACTOME_CYCLIN_E_ASSOCIATED_EVENTS_DURING_G1_S_TRANSITION_* | 24 | 1.79 | 8.21E-03 | 4.76E-02 |
| MIPS_17S_U2_SNRNP | 11 | 1.78 | 1.04E-02 | 4.86E-02 |
| MIPS_F1F0_ATP_SYNTHASE_MITOCHONDRIAL | 7 | 1.76 | 9.94E-03 | 5.87E-02 |
| REACTOME_SYNTHESIS_OF_DNA* | 26 | 1.76 | 1.11E-02 | 5.90E-02 |
| REACTOME_TRANSCRIPTION_COUPLED_NER_TC_NER | 8 | 1.75 | 1.01E-02 | 6.03E-02 |
| REACTOME_CELL_CYCLE_CHECKPOINTS* | 31 | 1.74 | 1.04E-02 | 6.49E-02 |
| REACTOME_TRANSPORT_OF_RIBONUCLEOPROTEINS_INTO_THE_HOST_NUCLEUS* | 9 | 1.74 | 2.05E-02 | 6.60E-02 |
| MIPS_PA700_20S_PA28_COMPLEX* | 21 | 1.73 | 1.67E-02 | 6.82E-02 |
| REACTOME_REGULATION_OF_MRNA_STABILITY_BY_PROTEINS_THAT_BIND_AU_RICH_ELEMENTS* | 36 | 1.72 | 6.38E-03 | 7.01E-02 |
| REACTOME_DESTABILIZATION_OF_MRNA_BY_AUF1_HNRNP_D0* | 25 | 1.72 | 6.45E-03 | 7.22E-02 |
| REACTOME_AUTODEGRADATION_OF_THE_E3_UBIQUITIN_LIGASE_COP1* | 23 | 1.72 | 1.99E-02 | 7.23E-02 |
| BIOCARTA_EIF_PATHWAY | 9 | 1.71 | 6.24E-03 | 7.34E-02 |
| REACTOME_MITOTIC_G1_G1_S_PHASES* | 30 | 1.71 | 8.33E-03 | 7.46E-02 |
| BIOCARTA_IGF1MTOR_PATHWAY | 10 | 1.70 | 2.94E-02 | 7.65E-02 |
| REACTOME_MTORC1_MEDIATED_SIGNALLING | 8 | 1.70 | 2.74E-02 | 7.74E-02 |
| REACTOME_SCFSKP2_MEDIATED_DEGRADATION_OF_P27_P21* | 22 | 1.69 | 1.77E-02 | 8.00E-02 |
| REACTOME_REGULATION_OF_GLUCOKINASE_BY_GLUCOKINASE_REGULATORY_PROTEIN | 9 | 1.69 | 1.86E-02 | 8.03E-02 |
| REACTOME_P53_DEPENDENT_G1_DNA_DAMAGE_RESPONSE* | 25 | 1.69 | 1.71E-02 | 8.07E-02 |
| REACTOME_INTERACTIONS_OF_VPR_WITH_HOST_CELLULAR_PROTEINS | 11 | 1.68 | 2.00E-03 | 8.23E-02 |
| REACTOME_EXTENSION_OF_TELOMERES | 5 | 1.68 | 1.77E-02 | 8.47E-02 |
| REACTOME_CELL_CYCLE* | 57 | 1.68 | 2.00E-03 | 8.50E-02 |
| REACTOME_DNA_REPAIR | 14 | 1.67 | 2.02E-02 | 9.03E-02 |
| REACTOME_AUTODEGRADATION_OF_CDH1_BY_CDH1_APC_C* | 24 | 1.66 | 1.08E-02 | 9.13E-02 |
| BIOCARTA_VEGF_PATHWAY | 12 | 1.66 | 2.88E-02 | 9.15E-02 |
| MIPS_PA28_20S_PROTEASOME* | 8 | 1.66 | 3.14E-02 | 9.19E-02 |
| PID_MYC_PATHWAY | 5 | 1.66 | 1.83E-02 | 9.25E-02 |
| REACTOME_FORMATION_OF_ATP_BY_CHEMIOSMOTIC_COUPLING | 6 | 1.65 | 2.61E-02 | 9.79E-02 |
| REACTOME_TRANSPORT_OF_MATURE_TRANSCRIPT_TO_CYTOPLASM* | 15 | 1.64 | 2.72E-02 | 1.02E-01 |
| REACTOME_TELOMERE_MAINTENANCE* | 5 | 1.63 | 1.88E-02 | 1.06E-01 |
| MIPS_28S_RIBOSOMAL_SUBUNIT_MITOCHONDRIAL | 11 | 1.63 | 2.43E-02 | 1.07E-01 |
| REACTOME_CDK_MEDIATED_PHOSPHORYLATION_AND_REMOVAL_OF_CDC6* | 22 | 1.62 | 3.26E-02 | 1.10E-01 |
| REACTOME_CROSS_PRESENTATION_OF_SOLUBLE_EXOGENOUS_ANTIGENS_ENDOSOMES* | 22 | 1.61 | 2.45E-02 | 1.14E-01 |
| KEGG_ONE_CARBON_POOL_BY_FOLATE | 7 | 1.61 | 4.63E-02 | 1.14E-01 |
| REACTOME_NUCLEOTIDE_EXCISION_REPAIR | 11 | 1.61 | 3.79E-02 | 1.14E-01 |
| REACTOME_SCF_BETA_TRCP_MEDIATED_DEGRADATION_OF_EMI1* | 22 | 1.61 | 2.29E-02 | 1.15E-01 |
| REACTOME_APC_C_CDC20_MEDIATED_DEGRADATION_OF_MITOTIC_PROTEINS* | 25 | 1.60 | 2.30E-02 | 1.16E-01 |
| MIPS_CDC5L_COMPLEX | 13 | 1.60 | 3.56E-02 | 1.16E-01 |
| KEGG_SPLICEOSOME | 42 | 1.60 | 2.76E-02 | 1.18E-01 |
| REACTOME_REGULATION_OF_MITOTIC_CELL_CYCLE* | 25 | 1.59 | 2.75E-02 | 1.19E-01 |
| REACTOME_M_G1_TRANSITION* | 24 | 1.59 | 2.73E-02 | 1.22E-01 |
| REACTOME_REGULATION_OF_APOPTOSIS* | 22 | 1.57 | 3.50E-02 | 1.30E-01 |
| REACTOME_GLOBAL_GENOMIC_NER_GG_NER | 9 | 1.57 | 4.90E-02 | 1.30E-01 |
| REACTOME_ORC1_REMOVAL_FROM_CHROMATIN* | 23 | 1.57 | 3.34E-02 | 1.30E-01 |
| REACTOME_FORMATION_OF_TRANSCRIPTION_COUPLED_NER_TC_NER_REPAIR_COMPLEX | 5 | 1.57 | 5.72E-02 | 1.30E-01 |
| KEGG_ALZHEIMERS_DISEASE | 53 | 1.57 | 1.98E-02 | 1.31E-01 |
| REACTOME_FORMATION_OF_INCISION_COMPLEX_IN_GG_NER | 7 | 1.56 | 4.53E-02 | 1.37E-01 |
| KEGG_NUCLEOTIDE_EXCISION_REPAIR | 12 | 1.56 | 3.25E-02 | 1.37E-01 |
| REACTOME_APC_C_CDH1_MEDIATED_DEGRADATION_OF_CDC20_AND_OTHER_APC_C_CDH1_TARGETED_PROTEINS_IN_LATE_MITOSIS_EARLY_G1* | 25 | 1.55 | 3.45E-02 | 1.39E-01 |
| REACTOME_SIGNALING_BY_WNT* | 26 | 1.55 | 3.94E-02 | 1.39E-01 |
| REACTOME_MRNA_SPLICING_MINOR_PATHWAY | 10 | 1.55 | 7.16E-02 | 1.40E-01 |
| REACTOME_TRANSPORT_OF_MATURE_MRNA_DERIVED_FROM_AN_INTRONLESS_TRANSCRIPT | 12 | 1.55 | 4.30E-02 | 1.41E-01 |
| KEGG_PROTEASOME* | 24 | 1.54 | 4.46E-02 | 1.42E-01 |
| REACTOME_METABOLISM_OF_NUCLEOTIDES | 31 | 1.53 | 3.29E-02 | 1.43E-01 |
| KEGG_OXIDATIVE_PHOSPHORYLATION | 59 | 1.54 | 1.28E-02 | 1.44E-01 |
| REACTOME_HOST_INTERACTIONS_OF_HIV_FACTORS* | 46 | 1.53 | 2.61E-02 | 1.44E-01 |
| MIPS_C_COMPLEX_SPLICEOSOME | 27 | 1.53 | 3.21E-02 | 1.44E-01 |
| KEGG_RNA_DEGRADATION | 16 | 1.53 | 4.91E-02 | 1.45E-01 |
| REACTOME_MRNA_SPLICING | 27 | 1.54 | 3.64E-02 | 1.45E-01 |
| REACTOME_DESTABILIZATION_OF_MRNA_BY_KSRP | 6 | 1.52 | 5.73E-02 | 1.49E-01 |
| REACTOME_P53_INDEPENDENT_G1_S_DNA_DAMAGE_CHECKPOINT* | 23 | 1.52 | 4.77E-02 | 1.50E-01 |
| BIOCARTA_MTOR_PATHWAY | 8 | 1.50 | 9.59E-02 | 1.63E-01 |
| REACTOME_CDT1_ASSOCIATION_WITH_THE_CDC6_ORC_ORIGIN_COMPLEX* | 23 | 1.50 | 5.13E-02 | 1.63E-01 |
| REACTOME_ASSEMBLY_OF_THE_PRE_REPLICATIVE_COMPLEX* | 23 | 1.49 | 4.56E-02 | 1.72E-01 |
| MIPS_SPLICEOSOME | 48 | 1.47 | 3.57E-02 | 1.85E-01 |
| REACTOME_DNA_REPLICATION* | 37 | 1.46 | 5.58E-02 | 1.91E-01 |
| REACTOME_ACTIVATION_OF_NF_KAPPAB_IN_B_CELLS* | 24 | 1.44 | 6.32E-02 | 2.15E-01 |
| REACTOME_PURINE_METABOLISM | 14 | 1.42 | 7.73E-02 | 2.31E-01 |
| REACTOME_CELL_CYCLE_MITOTIC* | 49 | 1.42 | 4.47E-02 | 2.31E-01 |
| MIPS_26S_PROTEASOME* | 12 | 1.42 | 1.08E-01 | 2.32E-01 |
| MIPS_18S_U11_U12_SNRNP | 9 | 1.41 | 1.14E-01 | 2.40E-01 |
| PID_ATR_PATHWAY | 6 | 1.40 | 1.20E-01 | 2.46E-01 |
| REACTOME_GLUCOSE_TRANSPORT | 12 | 1.39 | 1.16E-01 | 2.47E-01 |
| KEGG_PROTEIN_EXPORT | 10 | 1.40 | 1.19E-01 | 2.48E-01 |
| REACTOME_MITOTIC_M_M_G1_PHASES* | 34 | 1.39 | 8.02E-02 | 2.49E-01 |
| REACTOME_ER_PHAGOSOME_PATHWAY* | 26 | 1.39 | 8.13E-02 | 2.49E-01 |

*: Pathways overlapping with up-regulated pathways in the *xmrk*-induced zebrafish liver cancer.

**Down-regulated canonical pathways in the *Myc*-induced zebrafish liver cancer**

| NAME | SIZE | NES | p-val | FDR q-val |
| --- | --- | --- | --- | --- |
| REACTOME_STRIATED_MUSCLE_CONTRACTION | 7 | -2.08 | 0.00E+00 | 2.32E-02 |
| KEGG_LYSOSOME | 35 | -2.12 | 0.00E+00 | 2.50E-02 |
| REACTOME_MUSCLE_CONTRACTION | 11 | -2.13 | 0.00E+00 | 4.33E-02 |
| REACTOME_GPCR_DOWNSTREAM_SIGNALING | 23 | -1.82 | 7.77E-03 | 8.89E-02 |
| REACTOME_METABOLISM_OF_LIPIDS_AND_LIPOPROTEINS | 96 | -1.82 | 0.00E+00 | 9.19E-02 |
| REACTOME_PLATELET_ACTIVATION_SIGNALING_AND_AGGREGATION | 45 | -1.83 | 1.94E-03 | 9.35E-02 |
| REACTOME_TRANSFERRIN_ENDOCYTOSIS_AND_RECYCLING | 9 | -1.81 | 3.72E-03 | 9.40E-02 |
| REACTOME_SIGNALING_BY_GPCR | 31 | -1.84 | 1.88E-03 | 9.42E-02 |
| KEGG_NEUROACTIVE_LIGAND_RECEPTOR_INTERACTION | 10 | -1.80 | 1.15E-02 | 9.70E-02 |
| REACTOME_IRON_UPTAKE_AND_TRANSPORT | 14 | -1.83 | 9.47E-03 | 9.71E-02 |
| REACTOME_ALPHA_LINOLENIC_ACID_ALA_METABOLISM | 5 | -1.83 | 3.87E-03 | 9.78E-02 |
| REACTOME_LATENT_INFECTION_OF_HOMO_SAPIENS_WITH_MYCOBACTERIUM_TUBERCULOSIS | 9 | -1.80 | 1.10E-02 | 9.79E-02 |
| KEGG_ADIPOCYTOKINE_SIGNALING_PATHWAY | 13 | -1.85 | 7.89E-03 | 9.97E-02 |
| REACTOME_TRANSPORT_OF_GLUCOSE_AND_OTHER_SUGARS_BILE_SALTS_AND_ORGANIC_ACIDS_METAL_IONS_AND_AMINE_COMPOUNDS | 16 | -1.79 | 0.00E+00 | 1.03E-01 |
| KEGG_HYPERTROPHIC_CARDIOMYOPATHY_HCM | 11 | -1.85 | 7.86E-03 | 1.07E-01 |
| REACTOME_SYNTHESIS_SECRETION_AND_DEACYLATION_OF_GHRELIN | 5 | -1.77 | 9.40E-03 | 1.12E-01 |
| KEGG_DILATED_CARDIOMYOPATHY | 10 | -1.85 | 1.96E-03 | 1.13E-01 |
| REACTOME_HEMOSTASIS | 68 | -1.93 | 0.00E+00 | 1.17E-01 |
| REACTOME_G_ALPHA_S_SIGNALLING_EVENTS | 7 | -1.77 | 7.46E-03 | 1.17E-01 |
| KEGG_EPITHELIAL_CELL_SIGNALING_IN_HELICOBACTER_PYLORI_INFECTION | 15 | -1.86 | 3.80E-03 | 1.18E-01 |
| KEGG_VIBRIO_CHOLERAE_INFECTION | 14 | -1.91 | 1.88E-03 | 1.18E-01 |
| REACTOME_REGULATION_OF_INSULIN_SECRETION | 13 | -1.88 | 0.00E+00 | 1.25E-01 |
| REACTOME_MHC_CLASS_II_ANTIGEN_PRESENTATION | 19 | -1.75 | 7.95E-03 | 1.27E-01 |
| REACTOME_TRANSMEMBRANE_TRANSPORT_OF_SMALL_MOLECULES | 64 | -1.75 | 2.01E-03 | 1.29E-01 |
| REACTOME_INSULIN_RECEPTOR_RECYCLING | 8 | -1.86 | 1.87E-03 | 1.32E-01 |
| KEGG_COMPLEMENT_AND_COAGULATION_CASCADES | 21 | -1.88 | 4.02E-03 | 1.45E-01 |
| PID_ERBB1_INTERNALIZATION_PATHWAY | 6 | -1.72 | 9.12E-03 | 1.62E-01 |
| REACTOME_TRANSMISSION_ACROSS_CHEMICAL_SYNAPSES | 13 | -1.69 | 1.93E-02 | 1.62E-01 |
| KEGG_ARRHYTHMOGENIC_RIGHT_VENTRICULAR_CARDIOMYOPATHY_ARVC | 6 | -1.69 | 1.30E-02 | 1.65E-01 |
| REACTOME_INCRETIN_SYNTHESIS_SECRETION_AND_INACTIVATION | 5 | -1.68 | 1.54E-02 | 1.68E-01 |
| REACTOME_GPCR_LIGAND_BINDING | 15 | -1.69 | 1.72E-02 | 1.68E-01 |
| REACTOME_OPIOID_SIGNALLING | 7 | -1.68 | 1.40E-02 | 1.69E-01 |
| REACTOME_SIGNALING_BY_NOTCH | 17 | -1.68 | 1.74E-02 | 1.69E-01 |
| REACTOME_NEUROTRANSMITTER_RECEPTOR_BINDING_AND_DOWNSTREAM_TRANSMISSION_IN_THE_POSTSYNAPTIC_CELL | 7 | -1.70 | 9.58E-03 | 1.69E-01 |
| REACTOME_PRE_NOTCH_PROCESSING_IN_GOLGI | 6 | -1.70 | 1.00E-02 | 1.71E-01 |
| REACTOME_BILE_ACID_AND_BILE_SALT_METABOLISM | 6 | -1.67 | 1.93E-02 | 1.74E-01 |
| KEGG_PEROXISOME | 29 | -1.70 | 5.68E-03 | 1.74E-01 |
| REACTOME_TRANSCRIPTIONAL_ACTIVITY_OF_SMAD2_SMAD3_SMAD4_HETEROTRIMER | 12 | -1.66 | 2.88E-02 | 1.75E-01 |
| KEGG_GLUTATHIONE_METABOLISM | 14 | -1.67 | 1.16E-02 | 1.75E-01 |
| REACTOME_OXYGEN_DEPENDENT_PROLINE_HYDROXYLATION_OF_HYPOXIA_INDUCIBLE_FACTOR_ALPHA | 8 | -1.70 | 1.76E-02 | 1.76E-01 |
| PID_IL8CXCR2_PATHWAY | 7 | -1.65 | 1.97E-02 | 1.80E-01 |
| REACTOME_SYNTHESIS_OF_BILE_ACIDS_AND_BILE_SALTS | 6 | -1.65 | 1.51E-02 | 1.81E-01 |
| KEGG_VASOPRESSIN_REGULATED_WATER_REABSORPTION | 10 | -1.64 | 2.93E-02 | 1.85E-01 |
| REACTOME_TRANS_GOLGI_NETWORK_VESICLE_BUDDING | 15 | -1.64 | 2.10E-02 | 1.87E-01 |
| KEGG_SNARE_INTERACTIONS_IN_VESICULAR_TRANSPORT | 7 | -1.64 | 1.82E-02 | 1.88E-01 |
| PID_HEDGEHOG_GLIPATHWAY | 8 | -1.63 | 2.50E-02 | 1.96E-01 |
| REACTOME_EXTRACELLULAR_MATRIX_ORGANIZATION | 8 | -1.62 | 2.10E-02 | 2.01E-01 |
| KEGG_LEUKOCYTE_TRANSENDOTHELIAL_MIGRATION | 24 | -1.61 | 2.26E-02 | 2.04E-01 |
| REACTOME_NEURONAL_SYSTEM | 15 | -1.62 | 1.98E-02 | 2.05E-01 |
| KEGG_RETINOL_METABOLISM | 8 | -1.61 | 3.68E-02 | 2.06E-01 |
| BIOCARTA_RAB_PATHWAY | 6 | -1.61 | 3.17E-02 | 2.08E-01 |
| REACTOME_REGULATION_OF_INSULIN_SECRETION_BY_GLUCAGON_LIKE_PEPTIDE1 | 6 | -1.60 | 2.37E-02 | 2.25E-01 |
| REACTOME_LIPID_DIGESTION_MOBILIZATION_AND_TRANSPORT | 8 | -1.58 | 4.28E-02 | 2.26E-01 |
| REACTOME_RIG_I_MDA5_MEDIATED_INDUCTION_OF_IFN_ALPHA_BETA_PATHWAYS | 11 | -1.57 | 4.29E-02 | 2.27E-01 |
| KEGG_CHEMOKINE_SIGNALING_PATHWAY | 19 | -1.59 | 2.45E-02 | 2.28E-01 |
| REACTOME_PLATELET_HOMEOSTASIS | 8 | -1.59 | 2.58E-02 | 2.28E-01 |
| KEGG_FATTY_ACID_METABOLISM | 19 | -1.58 | 4.62E-02 | 2.29E-01 |
| BIOCARTA_EXTRINSIC_PATHWAY | 7 | -1.58 | 2.77E-02 | 2.29E-01 |
| REACTOME_INNATE_IMMUNE_SYSTEM | 40 | -1.57 | 2.00E-02 | 2.30E-01 |
| KEGG_PPAR_SIGNALING_PATHWAY | 20 | -1.56 | 2.64E-02 | 2.31E-01 |
| KEGG_JAK_STAT_SIGNALING_PATHWAY | 15 | -1.58 | 3.77E-02 | 2.31E-01 |
| PID_INTEGRIN1_PATHWAY | 8 | -1.58 | 4.03E-02 | 2.32E-01 |
| KEGG_LONG_TERM_DEPRESSION | 9 | -1.56 | 4.14E-02 | 2.34E-01 |
| BIOCARTA_AMI_PATHWAY | 7 | -1.58 | 3.31E-02 | 2.35E-01 |
| KEGG_PRIMARY_BILE_ACID_BIOSYNTHESIS | 5 | -1.56 | 2.57E-02 | 2.39E-01 |
