# Supplementary material for: Synergistic Induction of Potential Warburg Effect in Zebrafish Hepatocellular Carcinoma by Co-Transgenic Expression of Myc and xmrk Oncogenes
Source: PLoS One. 2015 Jul 6;10(7):e0132319. doi: 10.1371/journal.pone.0132319 (PMC4492623; doi:10.1371/journal.pone.0132319)
Supplement: S7 Table — (DOCX) [file pone.0132319.s008.docx]

**S7 Table. Differentially expressed canonical pathways in *xmrk*-induced liver tumors**

**Up-regulated canonical pathways in *xmrk*-induced liver tumors**

| NAME | SIZE | NES | p-val | FDR |
| --- | --- | --- | --- | --- |
| REACTOME_HOST_INTERACTIONS_OF_HIV_FACTORS* | 57 | 1.90 | 0.00E+00 | 9.40E-05 |
| REACTOME_G1_S_TRANSITION* | 46 | 1.86 | 0.00E+00 | 2.04E-04 |
| REACTOME_HIV_INFECTION | 79 | 1.70 | 0.00E+00 | 2.30E-03 |
| REACTOME_ANTIGEN_PROCESSING_CROSS_PRESENTATION | 35 | 1.69 | 0.00E+00 | 2.90E-03 |
| REACTOME_DNA_REPLICATION* | 62 | 1.95 | 0.00E+00 | 7.25E-03 |
| KEGG_PROTEASOME | 31 | 1.95 | 0.00E+00 | 7.88E-03 |
| REACTOME_SYNTHESIS_OF_SUBSTRATES_IN_N_GLYCAN_BIOSYTHESIS | 7 | 2.01 | 0.00E+00 | 8.67E-03 |
| REACTOME_MITOTIC_M_M_G1_PHASES* | 57 | 1.96 | 0.00E+00 | 9.23E-03 |
| REACTOME_M_G1_TRANSITION | 37 | 1.97 | 0.00E+00 | 1.24E-02 |
| MIPS_PA700_20S_PA28_COMPLEX* | 27 | 1.88 | 0.00E+00 | 1.29E-02 |
| REACTOME_CDK_MEDIATED_PHOSPHORYLATION_AND_REMOVAL_OF_CDC6* | 29 | 1.86 | 0.00E+00 | 1.38E-02 |
| REACTOME_MITOTIC_G1_G1_S_PHASES* | 49 | 1.86 | 0.00E+00 | 1.38E-02 |
| REACTOME_P53_DEPENDENT_G1_DNA_DAMAGE_RESPONSE* | 32 | 1.88 | 0.00E+00 | 1.41E-02 |
| REACTOME_ORC1_REMOVAL_FROM_CHROMATIN* | 34 | 1.90 | 0.00E+00 | 1.42E-02 |
| REACTOME_REGULATION_OF_APOPTOSIS* | 30 | 1.85 | 0.00E+00 | 1.42E-02 |
| REACTOME_VIF_MEDIATED_DEGRADATION_OF_APOBEC3G | 32 | 1.85 | 0.00E+00 | 1.47E-02 |
| REACTOME_SYNTHESIS_OF_DNA* | 41 | 1.88 | 0.00E+00 | 1.48E-02 |
| REACTOME_AUTODEGRADATION_OF_THE_E3_UBIQUITIN_LIGASE_COP1* | 30 | 1.86 | 0.00E+00 | 1.49E-02 |
| PID_A6B1_A6B4_INTEGRIN_PATHWAY | 14 | 1.85 | 1.04E-03 | 1.49E-02 |
| REACTOME_CDT1_ASSOCIATION_WITH_THE_CDC6_ORC_ORIGIN_COMPLEX* | 30 | 1.87 | 0.00E+00 | 1.51E-02 |
| REACTOME_CELL_CYCLE_CHECKPOINTS* | 45 | 1.85 | 0.00E+00 | 1.57E-02 |
| REACTOME_ASSEMBLY_OF_THE_PRE_REPLICATIVE_COMPLEX* | 33 | 1.88 | 0.00E+00 | 1.65E-02 |
| REACTOME_CROSS_PRESENTATION_OF_SOLUBLE_EXOGENOUS_ANTIGENS_ENDOSOMES* | 29 | 1.83 | 0.00E+00 | 1.72E-02 |
| BIOCARTA_CTCF_PATHWAY | 10 | 1.81 | 0.00E+00 | 1.84E-02 |
| REACTOME_P53_INDEPENDENT_G1_S_DNA_DAMAGE_CHECKPOINT* | 30 | 1.81 | 0.00E+00 | 1.84E-02 |
| REACTOME_CELL_CYCLE* | 96 | 1.82 | 0.00E+00 | 1.87E-02 |
| REACTOME_CELL_CYCLE_MITOTIC* | 83 | 1.82 | 0.00E+00 | 1.88E-02 |
| REACTOME_L1CAM_INTERACTIONS | 17 | 1.81 | 0.00E+00 | 1.91E-02 |
| REACTOME_S_PHASE* | 44 | 1.82 | 0.00E+00 | 1.95E-02 |
| REACTOME_REGULATION_OF_ORNITHINE_DECARBOXYLASE_ODC | 34 | 1.81 | 0.00E+00 | 1.96E-02 |
| REACTOME_CD28_CO_STIMULATION | 10 | 1.79 | 0.00E+00 | 2.22E-02 |
| REACTOME_SCF_BETA_TRCP_MEDIATED_DEGRADATION_OF_EMI1* | 30 | 1.79 | 0.00E+00 | 2.26E-02 |
| REACTOME_SCFSKP2_MEDIATED_DEGRADATION_OF_P27_P21* | 30 | 1.78 | 0.00E+00 | 2.49E-02 |
| REACTOME_SIGNALING_BY_THE_B_CELL_RECEPTOR_BCR | 50 | 1.78 | 0.00E+00 | 2.52E-02 |
| REACTOME_RECYCLING_PATHWAY_OF_L1 | 8 | 1.78 | 2.25E-03 | 2.54E-02 |
| MIPS_PA28_20S_PROTEASOME* | 11 | 1.77 | 4.41E-03 | 2.58E-02 |
| REACTOME_APC_C_CDC20_MEDIATED_DEGRADATION_OF_MITOTIC_PROTEINS* | 35 | 1.76 | 0.00E+00 | 2.59E-02 |
| REACTOME_SIGNALING_BY_WNT* | 35 | 1.76 | 0.00E+00 | 2.63E-02 |
| MIPS_26S_PROTEASOME* | 15 | 1.76 | 1.06E-03 | 2.64E-02 |
| REACTOME_REGULATION_OF_MITOTIC_CELL_CYCLE* | 38 | 1.76 | 0.00E+00 | 2.65E-02 |
| PID_CXCR4_PATHWAY | 20 | 1.76 | 2.06E-03 | 2.66E-02 |
| REACTOME_AXON_GUIDANCE | 36 | 1.77 | 0.00E+00 | 2.66E-02 |
| REACTOME_APC_C_CDH1_MEDIATED_DEGRADATION_OF_CDC20_AND_OTHER_APC_C_CDH1_TARGETED_PROTEINS_IN_LATE_MITOSIS_EARLY_G1* | 34 | 1.76 | 0.00E+00 | 2.70E-02 |
| ST_FAS_SIGNALING_PATHWAY | 12 | 1.75 | 1.08E-03 | 2.72E-02 |
| PID_FOXM1PATHWAY | 11 | 1.77 | 1.08E-03 | 2.73E-02 |
| REACTOME_COSTIMULATION_BY_THE_CD28_FAMILY | 16 | 1.74 | 3.13E-03 | 2.96E-02 |
| PID_GMCSF_PATHWAY | 11 | 1.74 | 3.27E-03 | 3.14E-02 |
| REACTOME_G2_M_CHECKPOINTS | 8 | 1.72 | 2.24E-03 | 3.58E-02 |
| REACTOME_DOWNSTREAM_SIGNALING_EVENTS_OF_B_CELL_RECEPTOR_BCR | 48 | 1.73 | 0.00E+00 | 3.58E-02 |
| REACTOME_SIGNAL_TRANSDUCTION_BY_L1 | 7 | 1.72 | 4.64E-03 | 3.67E-02 |
| KEGG_DNA_REPLICATION | 15 | 1.44 | 3.81E-02 | 3.70E-02 |
| REACTOME_NCAM_SIGNALING_FOR_NEURITE_OUT_GROWTH | 7 | 1.72 | 2.38E-03 | 3.72E-02 |
| REACTOME_CYCLIN_E_ASSOCIATED_EVENTS_DURING_G1_S_TRANSITION_* | 34 | 1.71 | 0.00E+00 | 3.97E-02 |
| MIPS_PA700_COMPLEX | 16 | 1.71 | 2.11E-03 | 4.00E-02 |
| REACTOME_THE_ROLE_OF_NEF_IN_HIV1_REPLICATION_AND_DISEASE_PATHOGENESIS | 11 | 1.71 | 3.23E-03 | 4.01E-02 |
| REACTOME_DESTABILIZATION_OF_MRNA_BY_AUF1_HNRNP_D0* | 33 | 1.71 | 0.00E+00 | 4.08E-02 |
| REACTOME_SIGNALING_BY_GPCR | 43 | 1.41 | 1.40E-02 | 4.40E-02 |
| REACTOME_ER_PHAGOSOME_PATHWAY* | 33 | 1.69 | 0.00E+00 | 4.94E-02 |
| REACTOME_ACTIVATION_OF_NF_KAPPAB_IN_B_CELLS* | 37 | 1.69 | 0.00E+00 | 4.99E-02 |
| REACTOME_IL_3_5_AND_GM_CSF_SIGNALING | 7 | 1.68 | 8.14E-03 | 5.06E-02 |
| REACTOME_G_ALPHA1213_SIGNALLING_EVENTS | 10 | 1.69 | 2.17E-03 | 5.06E-02 |
| REACTOME_PI3K_EVENTS_IN_ERBB2_SIGNALING | 12 | 1.39 | 6.99E-02 | 5.07E-02 |
| KEGG_CELL_CYCLE | 27 | 1.68 | 2.01E-03 | 5.10E-02 |
| BIOCARTA_G2_PATHWAY | 8 | 1.66 | 5.69E-03 | 5.77E-02 |
| PID_ILK_PATHWAY | 11 | 1.66 | 2.16E-03 | 5.83E-02 |
| BIOCARTA_UCALPAIN_PATHWAY | 9 | 1.66 | 9.03E-03 | 5.86E-02 |
| REACTOME_PRE_NOTCH_EXPRESSION_AND_PROCESSING | 10 | 1.67 | 4.36E-03 | 5.93E-02 |
| REACTOME_MITOTIC_PROMETAPHASE | 19 | 1.66 | 3.08E-03 | 6.12E-02 |
| REACTOME_AUTODEGRADATION_OF_CDH1_BY_CDH1_APC_C* | 31 | 1.66 | 0.00E+00 | 6.24E-02 |
| REACTOME_APOPTOSIS | 53 | 1.65 | 0.00E+00 | 6.30E-02 |
| REACTOME_DEPOSITION_OF_NEW_CENPA_CONTAINING_NUCLEOSOMES_AT_THE_CENTROMERE | 6 | 1.65 | 9.46E-03 | 6.52E-02 |
| REACTOME_TRANSPORT_OF_RIBONUCLEOPROTEINS_INTO_THE_HOST_NUCLEUS* | 9 | 1.36 | 8.95E-02 | 6.53E-02 |
| BIOCARTA_PROTEASOME_PATHWAY | 22 | 1.64 | 3.03E-03 | 6.70E-02 |
| REACTOME_REGULATION_OF_KIT_SIGNALING | 5 | 1.64 | 1.20E-02 | 6.72E-02 |
| BIOCARTA_GLEEVEC_PATHWAY | 7 | 1.64 | 8.14E-03 | 6.76E-02 |
| MIPS_20S_PROTEASOME | 9 | 1.63 | 1.11E-02 | 7.60E-02 |
| REACTOME_CELL_CELL_COMMUNICATION | 25 | 1.61 | 4.05E-03 | 8.13E-02 |
| REACTOME_SIGNALING_BY_PDGF | 19 | 1.62 | 8.32E-03 | 8.18E-02 |
| PID_SYNDECAN_1_PATHWAY | 9 | 1.62 | 1.33E-02 | 8.27E-02 |
| REACTOME_ACTIVATION_OF_ATR_IN_RESPONSE_TO_REPLICATION_STRESS | 7 | 1.62 | 9.26E-03 | 8.33E-02 |
| REACTOME_ACTIVATION_OF_THE_PRE_REPLICATIVE_COMPLEX | 7 | 1.62 | 9.39E-03 | 8.38E-02 |
| REACTOME_ADAPTIVE_IMMUNE_SYSTEM | 155 | 1.62 | 0.00E+00 | 8.50E-02 |
| REACTOME_HIV_LIFE_CYCLE | 36 | 1.32 | 6.27E-02 | 8.55E-02 |
| REACTOME_PI3K_EVENTS_IN_ERBB4_SIGNALING | 9 | 1.31 | 1.24E-01 | 9.01E-02 |
| REACTOME_PIP3_ACTIVATES_AKT_SIGNALING | 9 | 1.30 | 1.13E-01 | 9.32E-02 |
| KEGG_NATURAL_KILLER_CELL_MEDIATED_CYTOTOXICITY | 10 | 1.59 | 1.40E-02 | 1.05E-01 |
| REACTOME_GPVI_MEDIATED_ACTIVATION_CASCADE | 9 | 1.59 | 1.33E-02 | 1.06E-01 |
| REACTOME_STRIATED_MUSCLE_CONTRACTION | 10 | 1.59 | 8.68E-03 | 1.06E-01 |
| REACTOME_CELL_SURFACE_INTERACTIONS_AT_THE_VASCULAR_WALL | 19 | 1.58 | 7.25E-03 | 1.06E-01 |
| SA_B_CELL_RECEPTOR_COMPLEXES | 5 | 1.59 | 8.62E-03 | 1.07E-01 |
| PID_RAC1_REG_PATHWAY | 7 | 1.58 | 9.37E-03 | 1.07E-01 |
| BIOCARTA_GH_PATHWAY | 7 | 1.58 | 2.78E-02 | 1.09E-01 |
| BIOCARTA_AKAP95_PATHWAY | 5 | 1.58 | 1.50E-02 | 1.10E-01 |
| REACTOME_LOSS_OF_NLP_FROM_MITOTIC_CENTROSOMES | 12 | 1.57 | 1.49E-02 | 1.10E-01 |
| BIOCARTA_FCER1_PATHWAY | 7 | 1.58 | 1.28E-02 | 1.11E-01 |
| REACTOME_CELL_JUNCTION_ORGANIZATION | 17 | 1.57 | 8.32E-03 | 1.12E-01 |
| REACTOME_SIGNALING_BY_SCF_KIT | 21 | 1.57 | 2.05E-03 | 1.12E-01 |
| REACTOME_BIOSYNTHESIS_OF_THE_N_GLYCAN_PRECURSOR_DOLICHOL_LIPID_LINKED_OLIGOSACCHARIDE_LLO_AND_TRANSFER_TO_A_NASCENT_PROTEIN | 12 | 1.56 | 1.40E-02 | 1.19E-01 |
| REACTOME_PRE_NOTCH_PROCESSING_IN_GOLGI | 8 | 1.57 | 1.57E-02 | 1.19E-01 |
| MIPS_ALL_1_SUPERCOMPLEX | 14 | 1.56 | 9.49E-03 | 1.19E-01 |
| KEGG_CHEMOKINE_SIGNALING_PATHWAY | 31 | 1.55 | 2.02E-03 | 1.33E-01 |
| REACTOME_NEUROTRANSMITTER_RECEPTOR_BINDING_AND_DOWNSTREAM_TRANSMISSION_IN_THE_POSTSYNAPTIC_CELL | 12 | 1.55 | 1.06E-02 | 1.33E-01 |
| SIG_BCR_SIGNALING_PATHWAY | 10 | 1.54 | 1.54E-02 | 1.34E-01 |
| BIOCARTA_CHREBP2_PATHWAY | 9 | 1.54 | 1.22E-02 | 1.34E-01 |
| REACTOME_GAB1_SIGNALOSOME | 13 | 1.55 | 1.92E-02 | 1.34E-01 |
| REACTOME_NEF_MEDIATES_DOWN_MODULATION_OF_CELL_SURFACE_RECEPTORS_BY_RECRUITING_THEM_TO_CLATHRIN_ADAPTERS | 8 | 1.54 | 2.49E-02 | 1.34E-01 |
| BIOCARTA_MYOSIN_PATHWAY | 7 | 1.55 | 1.37E-02 | 1.35E-01 |
| PID_FAK_PATHWAY | 13 | 1.55 | 1.59E-02 | 1.35E-01 |
| PID_ERB_GENOMIC_PATHWAY | 6 | 1.54 | 1.31E-02 | 1.36E-01 |
| REACTOME_EARLY_PHASE_OF_HIV_LIFE_CYCLE | 5 | 1.25 | 1.61E-01 | 1.36E-01 |
| REACTOME_CHROMOSOME_MAINTENANCE* | 20 | 1.54 | 1.56E-02 | 1.39E-01 |
| REACTOME_IMMUNE_SYSTEM | 229 | 1.54 | 0.00E+00 | 1.39E-01 |
| REACTOME_GLYCOSPHINGOLIPID_METABOLISM | 7 | 1.53 | 2.30E-02 | 1.41E-01 |
| REACTOME_CELL_CELL_JUNCTION_ORGANIZATION | 12 | 1.52 | 2.25E-02 | 1.41E-01 |
| REACTOME_FACTORS_INVOLVED_IN_MEGAKARYOCYTE_DEVELOPMENT_AND_PLATELET_PRODUCTION | 19 | 1.52 | 1.54E-02 | 1.41E-01 |
| BIOCARTA_PTDINS_PATHWAY | 7 | 1.53 | 2.66E-02 | 1.41E-01 |
| REACTOME_MHC_CLASS_II_ANTIGEN_PRESENTATION | 28 | 1.53 | 8.12E-03 | 1.41E-01 |
| PID_IL5_PATHWAY | 5 | 1.53 | 2.02E-02 | 1.42E-01 |
| PID_INTEGRIN1_PATHWAY | 13 | 1.52 | 1.61E-02 | 1.42E-01 |
| PID_AURORA_B_PATHWAY* | 13 | 1.52 | 2.24E-02 | 1.43E-01 |
| REACTOME_MEMBRANE_TRAFFICKING | 44 | 1.52 | 2.00E-03 | 1.43E-01 |
| REACTOME_AQUAPORIN_MEDIATED_TRANSPORT | 5 | 1.53 | 1.87E-02 | 1.43E-01 |
| REACTOME_INTRINSIC_PATHWAY_FOR_APOPTOSIS | 8 | 1.52 | 2.78E-02 | 1.43E-01 |
| REACTOME_DNA_STRAND_ELONGATION | 10 | 1.51 | 2.20E-02 | 1.44E-01 |
| REACTOME_ASPARAGINE_N_LINKED_GLYCOSYLATION | 32 | 1.51 | 7.06E-03 | 1.44E-01 |
| PID_TRKRPATHWAY | 13 | 1.53 | 1.82E-02 | 1.44E-01 |
| REACTOME_THROMBIN_SIGNALLING_THROUGH_PROTEINASE_ACTIVATED_RECEPTORS_PARS | 5 | 1.52 | 2.76E-02 | 1.44E-01 |
| REACTOME_RECRUITMENT_OF_MITOTIC_CENTROSOME_PROTEINS_AND_COMPLEXES | 14 | 1.52 | 2.00E-02 | 1.44E-01 |
| REACTOME_E2F_MEDIATED_REGULATION_OF_DNA_REPLICATION | 8 | 1.52 | 2.82E-02 | 1.44E-01 |
| PID_ALK1PATHWAY | 9 | 1.51 | 2.81E-02 | 1.46E-01 |
| REACTOME_REGULATION_OF_WATER_BALANCE_BY_RENAL_AQUAPORINS | 5 | 1.51 | 2.77E-02 | 1.47E-01 |
| MIPS_LARGE_DROSHA_COMPLEX | 10 | 1.50 | 3.35E-02 | 1.48E-01 |
| REACTOME_REGULATION_OF_MRNA_STABILITY_BY_PROTEINS_THAT_BIND_AU_RICH_ELEMENTS* | 47 | 1.50 | 3.01E-03 | 1.49E-01 |
| BIOCARTA_GPCR_PATHWAY | 6 | 1.50 | 3.58E-02 | 1.50E-01 |
| REACTOME_CLASS_I_MHC_MEDIATED_ANTIGEN_PROCESSING_PRESENTATION | 92 | 1.51 | 1.00E-03 | 1.50E-01 |
| REACTOME_REGULATION_OF_INSULIN_SECRETION_BY_GLUCAGON_LIKE_PEPTIDE1 | 6 | 1.50 | 3.29E-02 | 1.50E-01 |
| PID_NETRIN_PATHWAY | 6 | 1.51 | 2.49E-02 | 1.51E-01 |
| BIOCARTA_MCALPAIN_PATHWAY | 6 | 1.49 | 2.72E-02 | 1.58E-01 |
| PID_TXA2PATHWAY | 17 | 1.49 | 1.68E-02 | 1.59E-01 |
| REACTOME_ANTIGEN_PROCESSING_UBIQUITINATION_PROTEASOME_DEGRADATION | 83 | 1.50 | 1.00E-03 | 1.59E-01 |
| BIOCARTA_FAS_PATHWAY | 10 | 1.49 | 2.93E-02 | 1.59E-01 |
| MIPS_SNF2H_COHESIN_NURD_COMPLEX | 6 | 1.48 | 3.20E-02 | 1.73E-01 |
| PID_ERBB2ERBB3PATHWAY | 7 | 1.48 | 3.66E-02 | 1.73E-01 |
| REACTOME_DEVELOPMENTAL_BIOLOGY | 69 | 1.48 | 1.00E-03 | 1.75E-01 |
| REACTOME_NEP_NS2_INTERACTS_WITH_THE_CELLULAR_EXPORT_MACHINERY* | 10 | 1.20 | 2.28E-01 | 1.75E-01 |
| REACTOME_SEMAPHORIN_INTERACTIONS | 10 | 1.48 | 3.38E-02 | 1.76E-01 |
| PID_TAP63PATHWAY | 11 | 1.48 | 2.96E-02 | 1.77E-01 |
| KEGG_B_CELL_RECEPTOR_SIGNALING_PATHWAY | 18 | 1.48 | 2.29E-02 | 1.78E-01 |
| KEGG_LONG_TERM_POTENTIATION | 10 | 1.47 | 3.37E-02 | 1.80E-01 |
| KEGG_NEUROTROPHIN_SIGNALING_PATHWAY | 27 | 1.47 | 1.73E-02 | 1.81E-01 |
| PID_NECTIN_PATHWAY | 7 | 1.47 | 2.74E-02 | 1.82E-01 |
| REACTOME_SMOOTH_MUSCLE_CONTRACTION | 7 | 1.47 | 5.11E-02 | 1.85E-01 |
| PID_EPHRINBREVPATHWAY | 5 | 1.47 | 5.04E-02 | 1.86E-01 |
| REACTOME_COLLAGEN_FORMATION | 6 | 1.47 | 3.95E-02 | 1.87E-01 |
| REACTOME_MITOTIC_G2_G2_M_PHASES | 15 | 1.46 | 3.63E-02 | 1.88E-01 |
| MIPS_HDAC1_ASSOCIATED_CORE_COMPLEX_CII | 6 | 1.46 | 4.53E-02 | 1.89E-01 |
| PID_GLYPICAN_1PATHWAY | 7 | 1.46 | 3.66E-02 | 1.91E-01 |
| REACTOME_DOWNSTREAM_SIGNALING_OF_ACTIVATED_FGFR | 18 | 1.46 | 2.50E-02 | 1.92E-01 |
| KEGG_COLORECTAL_CANCER | 14 | 1.45 | 2.98E-02 | 1.98E-01 |
| KEGG_THYROID_CANCER | 10 | 1.45 | 4.95E-02 | 1.98E-01 |
| REACTOME_DOWNSTREAM_SIGNAL_TRANSDUCTION | 16 | 1.45 | 2.73E-02 | 1.98E-01 |
| BIOCARTA_TPO_PATHWAY | 6 | 1.45 | 4.18E-02 | 1.99E-01 |
| BIOCARTA_TEL_PATHWAY | 6 | 1.45 | 4.20E-02 | 2.00E-01 |
| REACTOME_SIGNALING_BY_EGFR_IN_CANCER | 23 | 1.45 | 2.76E-02 | 2.02E-01 |
| REACTOME_G_ALPHA_Z_SIGNALLING_EVENTS | 6 | 1.45 | 5.33E-02 | 2.02E-01 |
| BIOCARTA_TCR_PATHWAY | 7 | 1.44 | 3.70E-02 | 2.04E-01 |
| MIPS_ANTI_HDAC2_COMPLEX | 7 | 1.44 | 5.48E-02 | 2.06E-01 |
| MIPS_RC_COMPLEX_DURING_G2_M_PHASE_OF_CELL_CYCLE | 5 | 1.43 | 4.94E-02 | 2.07E-01 |
| REACTOME_NGF_SIGNALLING_VIA_TRKA_FROM_THE_PLASMA_MEMBRANE | 28 | 1.43 | 2.64E-02 | 2.08E-01 |
| REACTOME_PROCESSIVE_SYNTHESIS_ON_THE_LAGGING_STRAND | 6 | 1.44 | 4.40E-02 | 2.08E-01 |
| REACTOME_REGULATION_OF_INSULIN_SECRETION | 14 | 1.43 | 3.93E-02 | 2.08E-01 |
| REACTOME_SIGNALING_BY_RHO_GTPASES | 17 | 1.43 | 4.69E-02 | 2.08E-01 |
| KEGG_ADHERENS_JUNCTION | 17 | 1.44 | 4.15E-02 | 2.08E-01 |
| MIPS_INO80_CHROMATIN_REMODELING_COMPLEX | 5 | 1.43 | 4.66E-02 | 2.08E-01 |
| REACTOME_CASPASE_MEDIATED_CLEAVAGE_OF_CYTOSKELETAL_PROTEINS | 6 | 1.43 | 5.79E-02 | 2.09E-01 |
| PID_EPHA_FWDPATHWAY | 5 | 1.43 | 5.82E-02 | 2.09E-01 |
| REACTOME_TELOMERE_MAINTENANCE* | 11 | 1.43 | 3.88E-02 | 2.09E-01 |
| REACTOME_OPIOID_SIGNALLING | 8 | 1.43 | 6.12E-02 | 2.09E-01 |
| PID_LKB1_PATHWAY | 12 | 1.43 | 4.06E-02 | 2.09E-01 |
| PID_PI3KCIPATHWAY | 13 | 1.44 | 4.13E-02 | 2.09E-01 |
| BIOCARTA_ARF_PATHWAY | 8 | 1.43 | 5.97E-02 | 2.09E-01 |
| PID_NEPHRIN_NEPH1_PATHWAY | 6 | 1.43 | 5.86E-02 | 2.09E-01 |
| REACTOME_GROWTH_HORMONE_RECEPTOR_SIGNALING | 9 | 1.43 | 4.93E-02 | 2.10E-01 |
| KEGG_CHRONIC_MYELOID_LEUKEMIA | 18 | 1.44 | 3.25E-02 | 2.10E-01 |
| MIPS_MULTISYNTHETASE_COMPLEX* | 6 | 1.43 | 5.15E-02 | 2.10E-01 |
| PID_PTP1BPATHWAY | 10 | 1.44 | 5.45E-02 | 2.10E-01 |
| REACTOME_SIGNALLING_BY_NGF | 41 | 1.43 | 1.50E-02 | 2.10E-01 |
| MIPS_LARC_COMPLEX | 6 | 1.42 | 7.25E-02 | 2.16E-01 |
| ST_INTEGRIN_SIGNALING_PATHWAY | 14 | 1.42 | 6.18E-02 | 2.17E-01 |
| BIOCARTA_TNFR1_PATHWAY | 8 | 1.42 | 5.61E-02 | 2.17E-01 |
| PID_IL3_PATHWAY | 10 | 1.42 | 6.39E-02 | 2.17E-01 |
| KEGG_N_GLYCAN_BIOSYNTHESIS | 20 | 1.42 | 3.88E-02 | 2.18E-01 |
| REACTOME_TRANSPORT_OF_MATURE_TRANSCRIPT_TO_CYTOPLASM* | 19 | 1.42 | 4.42E-02 | 2.18E-01 |
| KEGG_EPITHELIAL_CELL_SIGNALING_IN_HELICOBACTER_PYLORI_INFECTION | 20 | 1.41 | 4.00E-02 | 2.27E-01 |
| PID_RAC1_PATHWAY | 16 | 1.41 | 6.04E-02 | 2.29E-01 |
| REACTOME_RAP1_SIGNALLING | 5 | 1.40 | 7.70E-02 | 2.35E-01 |
| KEGG_T_CELL_RECEPTOR_SIGNALING_PATHWAY | 19 | 1.40 | 4.35E-02 | 2.37E-01 |
| REACTOME_SEMA4D_INDUCED_CELL_MIGRATION_AND_GROWTH_CONE_COLLAPSE | 7 | 1.40 | 7.36E-02 | 2.38E-01 |
| KEGG_DORSO_VENTRAL_AXIS_FORMATION | 6 | 1.40 | 6.13E-02 | 2.41E-01 |
| REACTOME_PLATELET_ACTIVATION_SIGNALING_AND_AGGREGATION | 57 | 1.40 | 1.20E-02 | 2.42E-01 |
| KEGG_BASAL_TRANSCRIPTION_FACTORS | 8 | 1.15 | 2.89E-01 | 2.49E-01 |
| BIOCARTA_P53_PATHWAY | 6 | 1.39 | 7.61E-02 | 2.50E-01 |

*: Pathways overlapping with up-regulated pathways in the *Myc*-induced zebrafish liver cancer.

**Down-regulated canonical pathways in *xmrk*-induced liver tumors**

| NAME | SIZE | NES | p-val | FDR |
| --- | --- | --- | --- | --- |
| KEGG_FATTY_ACID_METABOLISM | 20 | -3.47 | 0.00E+00 | 0.00E+00 |
| KEGG_TRYPTOPHAN_METABOLISM | 20 | -3.34 | 0.00E+00 | 0.00E+00 |
| KEGG_GLYCOLYSIS_GLUCONEOGENESIS | 22 | -3.06 | 0.00E+00 | 0.00E+00 |
| KEGG_PEROXISOME | 32 | -2.97 | 0.00E+00 | 0.00E+00 |
| KEGG_BIOSYNTHESIS_OF_UNSATURATED_FATTY_ACIDS | 9 | -2.84 | 0.00E+00 | 0.00E+00 |
| REACTOME_MITOCHONDRIAL_FATTY_ACID_BETA_OXIDATION | 10 | -2.41 | 0.00E+00 | 1.29E-05 |
| KEGG_PPAR_SIGNALING_PATHWAY | 24 | -2.56 | 0.00E+00 | 6.07E-04 |
| REACTOME_FORMATION_OF_THE_TERNARY_COMPLEX_AND_SUBSEQUENTLY_THE_43S_COMPLEX | 35 | -2.50 | 0.00E+00 | 6.19E-04 |
| REACTOME_PEROXISOMAL_LIPID_METABOLISM | 10 | -2.51 | 0.00E+00 | 7.07E-04 |
| KEGG_BETA_ALANINE_METABOLISM | 11 | -2.43 | 0.00E+00 | 1.32E-03 |
| MIPS_60S_RIBOSOMAL_SUBUNIT_CYTOPLASMIC | 39 | -1.73 | 0.00E+00 | 1.88E-03 |
| REACTOME_BILE_ACID_AND_BILE_SALT_METABOLISM | 6 | -2.32 | 0.00E+00 | 1.96E-03 |
| KEGG_VALINE_LEUCINE_AND_ISOLEUCINE_DEGRADATION | 25 | -2.32 | 0.00E+00 | 2.13E-03 |
| BIOCARTA_LONGEVITY_PATHWAY | 6 | -2.33 | 0.00E+00 | 2.32E-03 |
| REACTOME_SYNTHESIS_OF_BILE_ACIDS_AND_BILE_SALTS | 6 | -2.34 | 0.00E+00 | 2.43E-03 |
| REACTOME_COMMON_PATHWAY | 6 | -1.64 | 1.81E-02 | 2.97E-03 |
| KEGG_STEROID_HORMONE_BIOSYNTHESIS | 9 | -2.27 | 0.00E+00 | 3.10E-03 |
| KEGG_PYRUVATE_METABOLISM | 18 | -2.26 | 0.00E+00 | 3.35E-03 |
| KEGG_METABOLISM_OF_XENOBIOTICS_BY_CYTOCHROME_P450 | 10 | -2.23 | 0.00E+00 | 3.61E-03 |
| KEGG_COMPLEMENT_AND_COAGULATION_CASCADES | 23 | -2.19 | 0.00E+00 | 4.45E-03 |
| REACTOME_ALPHA_LINOLENIC_ACID_ALA_METABOLISM | 5 | -2.19 | 0.00E+00 | 4.65E-03 |
| KEGG_PANTOTHENATE_AND_COA_BIOSYNTHESIS | 8 | -2.18 | 0.00E+00 | 4.96E-03 |
| KEGG_GLYCINE_SERINE_AND_THREONINE_METABOLISM | 19 | -2.17 | 2.86E-02 | 5.33E-03 |
| BIOCARTA_INTRINSIC_PATHWAY | 8 | -2.10 | 0.00E+00 | 7.99E-03 |
| REACTOME_CITRIC_ACID_CYCLE_TCA_CYCLE | 16 | -1.45 | 4.65E-02 | 8.01E-03 |
| KEGG_BUTANOATE_METABOLISM | 14 | -2.10 | 0.00E+00 | 8.03E-03 |
| REACTOME_GLUCONEOGENESIS | 16 | -2.08 | 0.00E+00 | 8.13E-03 |
| REACTOME_SYNTHESIS_OF_BILE_ACIDS_AND_BILE_SALTS_VIA_7ALPHA_HYDROXYCHOLESTEROL | 5 | -2.11 | 0.00E+00 | 8.24E-03 |
| KEGG_PROPANOATE_METABOLISM | 17 | -2.11 | 0.00E+00 | 8.44E-03 |
| KEGG_PRIMARY_BILE_ACID_BIOSYNTHESIS | 5 | -2.08 | 5.24E-03 | 8.45E-03 |
| KEGG_DRUG_METABOLISM_CYTOCHROME_P450 | 9 | -2.06 | 0.00E+00 | 9.09E-03 |
| KEGG_LIMONENE_AND_PINENE_DEGRADATION | 6 | -2.05 | 0.00E+00 | 9.30E-03 |
| MIPS_40S_RIBOSOMAL_SUBUNIT_CYTOPLASMIC | 25 | -1.98 | 0.00E+00 | 1.39E-02 |
| REACTOME_ACTIVATION_OF_THE_MRNA_UPON_BINDING_OF_THE_CAP_BINDING_COMPLEX_AND_EIFS_AND_SUBSEQUENT_BINDING_TO_43S | 39 | -1.96 | 0.00E+00 | 1.57E-02 |
| MIPS_EIF3_COMPLEX | 7 | -1.95 | 0.00E+00 | 1.59E-02 |
| REACTOME_RESPIRATORY_ELECTRON_TRANSPORT_ATP_SYNTHESIS_BY_CHEMIOSMOTIC_COUPLING_AND_HEAT_PRODUCTION_BY_UNCOUPLING_PROTEINS_ | 49 | -1.96 | 0.00E+00 | 1.61E-02 |
| REACTOME_GAMMA_CARBOXYLATION_TRANSPORT_AND_AMINO_TERMINAL_CLEAVAGE_OF_PROTEINS | 5 | -1.92 | 1.97E-02 | 1.84E-02 |
| REACTOME_TRYPTOPHAN_CATABOLISM | 5 | -1.92 | 4.81E-03 | 1.87E-02 |
| REACTOME_FORMATION_OF_FIBRIN_CLOT_CLOTTING_CASCADE | 12 | -1.91 | 0.00E+00 | 1.92E-02 |
| PID_INTEGRIN2_PATHWAY | 7 | -1.86 | 0.00E+00 | 2.24E-02 |
| REACTOME_FORMATION_OF_ATP_BY_CHEMIOSMOTIC_COUPLING | 6 | -1.86 | 2.05E-02 | 2.24E-02 |
| KEGG_PENTOSE_PHOSPHATE_PATHWAY | 9 | -1.86 | 8.13E-03 | 2.25E-02 |
| KEGG_ARACHIDONIC_ACID_METABOLISM | 13 | -1.87 | 1.61E-02 | 2.28E-02 |
| REACTOME_RESPIRATORY_ELECTRON_TRANSPORT | 41 | -1.85 | 0.00E+00 | 2.29E-02 |
| KEGG_LYSINE_DEGRADATION | 15 | -1.83 | 0.00E+00 | 2.38E-02 |
| REACTOME_INTRINSIC_PATHWAY | 6 | -1.83 | 6.54E-03 | 2.41E-02 |
| KEGG_CITRATE_CYCLE_TCA_CYCLE | 21 | -1.75 | 0.00E+00 | 3.39E-02 |
| KEGG_ALPHA_LINOLENIC_ACID_METABOLISM | 7 | -1.75 | 1.46E-02 | 3.47E-02 |
| BIOCARTA_AMI_PATHWAY | 8 | -1.74 | 0.00E+00 | 3.48E-02 |
| BIOCARTA_FIBRINOLYSIS_PATHWAY | 5 | -1.73 | 5.18E-03 | 3.65E-02 |
| KEGG_ASCORBATE_AND_ALDARATE_METABOLISM | 5 | -1.68 | 3.09E-02 | 4.60E-02 |
| KEGG_RETINOL_METABOLISM | 12 | -1.66 | 0.00E+00 | 5.16E-02 |
| REACTOME_TRIGLYCERIDE_BIOSYNTHESIS | 9 | -1.63 | 9.09E-03 | 5.86E-02 |
| KEGG_PENTOSE_AND_GLUCURONATE_INTERCONVERSIONS | 6 | -1.61 | 6.58E-03 | 6.26E-02 |
| BIOCARTA_NDKDYNAMIN_PATHWAY | 5 | -1.61 | 3.02E-02 | 6.33E-02 |
| REACTOME_BIOLOGICAL_OXIDATIONS | 31 | -1.60 | 0.00E+00 | 6.46E-02 |
| REACTOME_PTM_GAMMA_CARBOXYLATION_HYPUSINE_FORMATION_AND_ARYLSULFATASE_ACTIVATION | 8 | -1.58 | 1.67E-02 | 7.21E-02 |
| REACTOME_CLASS_A1_RHODOPSIN_LIKE_RECEPTORS | 13 | -0.89 | 7.01E-01 | 8.08E-02 |
| BIOCARTA_EXTRINSIC_PATHWAY | 8 | -1.55 | 2.75E-02 | 8.24E-02 |
| KEGG_NEUROACTIVE_LIGAND_RECEPTOR_INTERACTION | 11 | -1.54 | 7.32E-02 | 8.51E-02 |
| REACTOME_BRANCHED_CHAIN_AMINO_ACID_CATABOLISM | 9 | -1.54 | 4.17E-02 | 8.60E-02 |
| REACTOME_VITAMIN_B5_PANTOTHENATE_METABOLISM | 5 | -1.52 | 2.12E-02 | 8.94E-02 |
| REACTOME_BMAL1_CLOCK_NPAS2_ACTIVATES_CIRCADIAN_EXPRESSION | 7 | -0.84 | 6.78E-01 | 9.20E-02 |
| BIOCARTA_ETC_PATHWAY | 6 | -1.52 | 4.17E-02 | 9.26E-02 |
| KEGG_TYROSINE_METABOLISM | 12 | -1.50 | 6.76E-02 | 9.90E-02 |
| PID_HNF3BPATHWAY | 25 | -1.50 | 0.00E+00 | 9.90E-02 |
| REACTOME_PEPTIDE_LIGAND_BINDING_RECEPTORS | 7 | -0.79 | 8.35E-01 | 9.98E-02 |
| KEGG_LINOLEIC_ACID_METABOLISM | 6 | -1.46 | 6.62E-02 | 1.18E-01 |
| KEGG_PHENYLALANINE_METABOLISM | 7 | -1.45 | 5.80E-02 | 1.19E-01 |
| REACTOME_GLUCOSE_METABOLISM | 30 | -1.42 | 0.00E+00 | 1.38E-01 |
| REACTOME_METABOLISM_OF_PORPHYRINS | 9 | -1.39 | 7.45E-02 | 1.56E-01 |
| MIPS_TNF_ALPHA_NF_KAPPA_B_SIGNALING_COMPLEX_6 | 10 | -1.39 | 9.88E-02 | 1.57E-01 |
| REACTOME_GLYCEROPHOSPHOLIPID_BIOSYNTHESIS | 20 | -1.38 | 3.70E-02 | 1.58E-01 |
| KEGG_DRUG_METABOLISM_OTHER_ENZYMES | 11 | -1.35 | 6.49E-02 | 1.85E-01 |
| REACTOME_ABC_FAMILY_PROTEINS_MEDIATED_TRANSPORT | 5 | -1.32 | 1.22E-01 | 2.12E-01 |
| KEGG_GLYCEROLIPID_METABOLISM | 12 | -1.30 | 1.34E-01 | 2.21E-01 |
| REACTOME_PYRUVATE_METABOLISM | 8 | -1.30 | 1.53E-01 | 2.21E-01 |
| MIPS_F1F0_ATP_SYNTHASE_MITOCHONDRIAL | 7 | -1.29 | 1.60E-01 | 2.27E-01 |
| KEGG_STARCH_AND_SUCROSE_METABOLISM | 9 | -1.29 | 1.61E-01 | 2.30E-01 |
| REACTOME_TRANSPORT_OF_INORGANIC_CATIONS_ANIONS_AND_AMINO_ACIDS_OLIGOPEPTIDES | 13 | -1.28 | 1.67E-01 | 2.32E-01 |
| REACTOME_PYRIMIDINE_METABOLISM | 8 | -1.26 | 1.27E-01 | 2.47E-01 |
