# Supplementary material for: Synergistic Induction of Potential Warburg Effect in Zebrafish Hepatocellular Carcinoma by Co-Transgenic Expression of Myc and xmrk Oncogenes
Source: PLoS One. 2015 Jul 6;10(7):e0132319. doi: 10.1371/journal.pone.0132319 (PMC4492623; doi:10.1371/journal.pone.0132319)
Supplement: S8 Table — (DOCX) [file pone.0132319.s009.docx]

**S8 Table. Differentially expressed canonical pathways in *Myc*/*xmrk-*induced liver tumors**

**Upregulated canonical pathways in *Myc*/*xmrk-*induced liver tumors**

| NAME | SIZE | NES | p-val | FDR |
| --- | --- | --- | --- | --- |
| REACTOME_CHROMOSOME_MAINTENANCE | 27 | 2.21 | 0.00E+00 | 0.00E+00 |
| REACTOME_PROCESSIVE_SYNTHESIS_ON_THE_LAGGING_STRAND | 7 | 2.14 | 0.00E+00 | 4.64E-04 |
| REACTOME_TELOMERE_MAINTENANCE | 16 | 2.08 | 0.00E+00 | 1.24E-03 |
| KEGG_PROTEASOME | 32 | 2.03 | 0.00E+00 | 2.09E-03 |
| REACTOME_AUTODEGRADATION_OF_THE_E3_UBIQUITIN_LIGASE_COP1 | 31 | 2.01 | 0.00E+00 | 2.78E-03 |
| REACTOME_EXTENSION_OF_TELOMERES | 14 | 2.02 | 0.00E+00 | 2.79E-03 |
| REACTOME_P53_DEPENDENT_G1_DNA_DAMAGE_RESPONSE | 33 | 2.01 | 0.00E+00 | 3.02E-03 |
| REACTOME_MITOTIC_M_M_G1_PHASES | 65 | 2.01 | 0.00E+00 | 3.25E-03 |
| REACTOME_REGULATION_OF_APOPTOSIS | 31 | 2.01 | 0.00E+00 | 3.31E-03 |
| MIPS_PA700_20S_PA28_COMPLEX | 29 | 1.99 | 1.01E-03 | 3.33E-03 |
| REACTOME_CROSS_PRESENTATION_OF_SOLUBLE_EXOGENOUS_ANTIGENS_ENDOSOMES | 30 | 1.99 | 0.00E+00 | 3.37E-03 |
| REACTOME_M_G1_TRANSITION | 41 | 1.99 | 0.00E+00 | 3.62E-03 |
| REACTOME_DEPOSITION_OF_NEW_CENPA_CONTAINING_NUCLEOSOMES_AT_THE_CENTROMERE | 8 | 1.97 | 2.30E-03 | 3.99E-03 |
| REACTOME_DNA_REPLICATION | 72 | 1.97 | 0.00E+00 | 4.01E-03 |
| REACTOME_CDT1_ASSOCIATION_WITH_THE_CDC6_ORC_ORIGIN_COMPLEX | 31 | 1.96 | 0.00E+00 | 4.08E-03 |
| REACTOME_P53_INDEPENDENT_G1_S_DNA_DAMAGE_CHECKPOINT | 31 | 1.97 | 0.00E+00 | 4.21E-03 |
| REACTOME_REMOVAL_OF_THE_FLAP_INTERMEDIATE_FROM_THE_C_STRAND | 5 | 1.97 | 0.00E+00 | 4.23E-03 |
| REACTOME_S_PHASE | 51 | 1.95 | 0.00E+00 | 4.53E-03 |
| REACTOME_APC_C_CDH1_MEDIATED_DEGRADATION_OF_CDC20_AND_OTHER_APC_C_CDH1_TARGETED_PROTEINS_IN_LATE_MITOSIS_EARLY_G1 | 41 | 1.94 | 0.00E+00 | 5.21E-03 |
| REACTOME_HOST_INTERACTIONS_OF_HIV_FACTORS | 61 | 1.94 | 0.00E+00 | 5.26E-03 |
| REACTOME_SCF_BETA_TRCP_MEDIATED_DEGRADATION_OF_EMI1 | 31 | 1.93 | 0.00E+00 | 5.27E-03 |
| REACTOME_SYNTHESIS_OF_DNA | 47 | 1.94 | 0.00E+00 | 5.30E-03 |
| REACTOME_CELL_CYCLE | 121 | 1.93 | 0.00E+00 | 5.37E-03 |
| REACTOME_ASSEMBLY_OF_THE_PRE_REPLICATIVE_COMPLEX | 36 | 1.94 | 0.00E+00 | 5.42E-03 |
| REACTOME_DESTABILIZATION_OF_MRNA_BY_AUF1_HNRNP_D0 | 34 | 1.93 | 0.00E+00 | 5.49E-03 |
| REACTOME_INTRINSIC_PATHWAY_FOR_APOPTOSIS | 6 | 1.93 | 0.00E+00 | 5.59E-03 |
| REACTOME_ANTIGEN_PROCESSING_CROSS_PRESENTATION | 35 | 1.92 | 0.00E+00 | 5.75E-03 |
| REACTOME_CDK_MEDIATED_PHOSPHORYLATION_AND_REMOVAL_OF_CDC6 | 31 | 1.93 | 0.00E+00 | 5.75E-03 |
| REACTOME_APOPTOSIS | 49 | 1.90 | 0.00E+00 | 7.04E-03 |
| REACTOME_REGULATION_OF_ORNITHINE_DECARBOXYLASE_ODC | 35 | 1.90 | 0.00E+00 | 7.09E-03 |
| REACTOME_ER_PHAGOSOME_PATHWAY | 34 | 1.89 | 0.00E+00 | 7.41E-03 |
| REACTOME_REGULATION_OF_MITOTIC_CELL_CYCLE | 46 | 1.89 | 0.00E+00 | 7.44E-03 |
| REACTOME_CYTOSOLIC_TRNA_AMINOACYLATION | 15 | 1.89 | 0.00E+00 | 7.61E-03 |
| MIPS_ALL_1_SUPERCOMPLEX | 15 | 1.89 | 0.00E+00 | 7.83E-03 |
| REACTOME_SCFSKP2_MEDIATED_DEGRADATION_OF_P27_P21 | 32 | 1.88 | 0.00E+00 | 7.94E-03 |
| PID_AURORA_B_PATHWAY | 15 | 1.87 | 0.00E+00 | 9.63E-03 |
| REACTOME_CELL_CYCLE_CHECKPOINTS | 56 | 1.86 | 0.00E+00 | 1.04E-02 |
| REACTOME_LAGGING_STRAND_SYNTHESIS | 10 | 1.86 | 1.11E-03 | 1.06E-02 |
| REACTOME_DNA_STRAND_ELONGATION | 15 | 1.85 | 1.05E-03 | 1.12E-02 |
| REACTOME_ORC1_REMOVAL_FROM_CHROMATIN | 37 | 1.84 | 0.00E+00 | 1.22E-02 |
| REACTOME_APC_C_CDC20_MEDIATED_DEGRADATION_OF_MITOTIC_PROTEINS | 40 | 1.84 | 1.00E-03 | 1.29E-02 |
| MIPS_PA700_COMPLEX | 16 | 1.84 | 0.00E+00 | 1.32E-02 |
| REACTOME_AUTODEGRADATION_OF_CDH1_BY_CDH1_APC_C | 36 | 1.83 | 0.00E+00 | 1.35E-02 |
| REACTOME_MITOTIC_G1_G1_S_PHASES | 54 | 1.83 | 0.00E+00 | 1.40E-02 |
| REACTOME_CYCLIN_E_ASSOCIATED_EVENTS_DURING_G1_S_TRANSITION_ | 36 | 1.83 | 0.00E+00 | 1.42E-02 |
| PID_MYC_PATHWAY | 9 | 1.82 | 0.00E+00 | 1.45E-02 |
| REACTOME_KINESINS | 5 | 1.82 | 0.00E+00 | 1.48E-02 |
| REACTOME_CELL_CYCLE_MITOTIC | 104 | 1.81 | 0.00E+00 | 1.61E-02 |
| REACTOME_VIF_MEDIATED_DEGRADATION_OF_APOBEC3G | 33 | 1.81 | 0.00E+00 | 1.73E-02 |
| REACTOME_ACTIVATION_OF_NF_KAPPAB_IN_B_CELLS | 35 | 1.78 | 0.00E+00 | 2.08E-02 |
| REACTOME_ACTIVATION_OF_THE_PRE_REPLICATIVE_COMPLEX | 10 | 1.79 | 0.00E+00 | 2.10E-02 |
| KEGG_AMINOACYL_TRNA_BIOSYNTHESIS | 15 | 1.78 | 0.00E+00 | 2.11E-02 |
| MIPS_26S_PROTEASOME | 17 | 1.78 | 1.03E-03 | 2.15E-02 |
| REACTOME_SIGNALING_BY_WNT | 36 | 1.78 | 0.00E+00 | 2.20E-02 |
| REACTOME_HIV_INFECTION | 88 | 1.78 | 0.00E+00 | 2.24E-02 |
| MIPS_SMN_COMPLEX | 6 | 1.76 | 1.22E-03 | 2.71E-02 |
| KEGG_HOMOLOGOUS_RECOMBINATION | 7 | 1.76 | 0.00E+00 | 2.77E-02 |
| KEGG_SPLICEOSOME | 67 | 1.75 | 0.00E+00 | 2.88E-02 |
| REACTOME_G1_S_TRANSITION | 50 | 1.75 | 0.00E+00 | 2.90E-02 |
| REACTOME_TRNA_AMINOACYLATION | 17 | 1.75 | 3.11E-03 | 2.97E-02 |
| REACTOME_FACTORS_INVOLVED_IN_MEGAKARYOCYTE_DEVELOPMENT_AND_PLATELET_PRODUCTION | 17 | 1.74 | 1.04E-03 | 3.06E-02 |
| KEGG_ALANINE_ASPARTATE_AND_GLUTAMATE_METABOLISM | 11 | 1.74 | 4.36E-03 | 3.10E-02 |
| REACTOME_PROCESSING_OF_CAPPED_INTRON_CONTAINING_PRE_MRNA | 62 | 1.73 | 0.00E+00 | 3.61E-02 |
| MIPS_EMERIN_COMPLEX_25 | 10 | 1.73 | 3.30E-03 | 3.65E-02 |
| MIPS_28S_RIBOSOMAL_SUBUNIT_MITOCHONDRIAL | 18 | 1.43 | 4.96E-02 | 3.98E-02 |
| REACTOME_REGULATION_OF_MRNA_STABILITY_BY_PROTEINS_THAT_BIND_AU_RICH_ELEMENTS | 50 | 1.72 | 0.00E+00 | 4.03E-02 |
| REACTOME_RESOLUTION_OF_AP_SITES_VIA_THE_MULTIPLE_NUCLEOTIDE_PATCH_REPLACEMENT_PATHWAY | 5 | 1.71 | 0.00E+00 | 4.23E-02 |
| MIPS_55S_RIBOSOME_MITOCHONDRIAL | 46 | 1.42 | 1.60E-02 | 4.26E-02 |
| MIPS_C_COMPLEX_SPLICEOSOME | 41 | 1.71 | 0.00E+00 | 4.32E-02 |
| REACTOME_AMINO_ACID_SYNTHESIS_AND_INTERCONVERSION_TRANSAMINATION | 5 | 1.71 | 9.55E-03 | 4.33E-02 |
| MIPS_18S_U11_U12_SNRNP | 11 | 1.70 | 1.08E-03 | 4.36E-02 |
| REACTOME_BASE_EXCISION_REPAIR | 5 | 1.70 | 3.66E-03 | 4.39E-02 |
| REACTOME_METABOLISM_OF_NON_CODING_RNA | 23 | 1.70 | 3.04E-03 | 4.59E-02 |
| REACTOME_TRANSCRIPTION_COUPLED_NER_TC_NER | 20 | 1.69 | 3.08E-03 | 4.87E-02 |
| REACTOME_MRNA_PROCESSING | 68 | 1.69 | 0.00E+00 | 4.92E-02 |
| REACTOME_MRNA_SPLICING_MINOR_PATHWAY | 18 | 1.69 | 3.10E-03 | 4.93E-02 |
| KEGG_DNA_REPLICATION | 20 | 1.69 | 5.10E-03 | 4.95E-02 |
| MIPS_PA28_20S_PROTEASOME | 13 | 1.69 | 8.58E-03 | 4.98E-02 |
| MIPS_17S_U2_SNRNP | 18 | 1.68 | 3.14E-03 | 5.22E-02 |
| REACTOME_DOWNSTREAM_SIGNALING_EVENTS_OF_B_CELL_RECEPTOR_BCR | 43 | 1.67 | 1.00E-03 | 5.48E-02 |
| REACTOME_MITOTIC_PROMETAPHASE | 23 | 1.67 | 4.07E-03 | 5.62E-02 |
| REACTOME_SIGNALING_BY_THE_B_CELL_RECEPTOR_BCR | 46 | 1.67 | 1.00E-03 | 5.63E-02 |
| MIPS_SPLICEOSOME | 79 | 1.67 | 0.00E+00 | 5.71E-02 |
| REACTOME_MRNA_SPLICING | 48 | 1.66 | 1.00E-03 | 5.72E-02 |
| MIPS_MULTISYNTHETASE_COMPLEX | 6 | 1.66 | 1.20E-02 | 5.94E-02 |
| REACTOME_HOMOLOGOUS_RECOMBINATION_REPAIR_OF_REPLICATION_INDEPENDENT_DOUBLE_STRAND_BREAKS | 5 | 1.65 | 9.84E-03 | 6.27E-02 |
| PID_P38ALPHABETAPATHWAY | 8 | 1.65 | 1.48E-02 | 6.47E-02 |
| REACTOME_MITOCHONDRIAL_PROTEIN_IMPORT | 32 | 1.63 | 3.02E-03 | 7.71E-02 |
| PID_HDAC_CLASSI_PATHWAY | 21 | 1.63 | 7.18E-03 | 7.87E-02 |
| REACTOME_MEIOSIS | 13 | 1.63 | 1.06E-02 | 7.88E-02 |
| BIOCARTA_PROTEASOME_PATHWAY | 24 | 1.62 | 4.07E-03 | 7.99E-02 |
| REACTOME_RNA_POL_I_TRANSCRIPTION | 11 | 1.62 | 1.20E-02 | 8.34E-02 |
| BIOCARTA_ARF_PATHWAY | 8 | 1.62 | 2.04E-02 | 8.50E-02 |
| MIPS_H2AX_COMPLEX_ISOLATED_FROM_CELLS_WITHOUT_IR_EXPOSURE | 6 | 1.61 | 8.37E-03 | 8.52E-02 |
| REACTOME_NUCLEOTIDE_EXCISION_REPAIR | 23 | 1.61 | 5.09E-03 | 8.54E-02 |
| REACTOME_NEP_NS2_INTERACTS_WITH_THE_CELLULAR_EXPORT_MACHINERY | 13 | 1.61 | 1.39E-02 | 9.07E-02 |
| REACTOME_DOWNREGULATION_OF_TGF_BETA_RECEPTOR_SIGNALING | 6 | 1.60 | 1.19E-02 | 9.23E-02 |
| MIPS_INO80_CHROMATIN_REMODELING_COMPLEX | 7 | 1.60 | 1.28E-02 | 9.42E-02 |
| REACTOME_INTERACTIONS_OF_VPR_WITH_HOST_CELLULAR_PROTEINS | 14 | 1.59 | 9.47E-03 | 9.64E-02 |
| MIPS_CDC5L_COMPLEX | 17 | 1.59 | 9.40E-03 | 9.68E-02 |
| REACTOME_TRANSPORT_OF_MATURE_TRANSCRIPT_TO_CYTOPLASM | 25 | 1.60 | 4.04E-03 | 9.71E-02 |
| MIPS_LARC_COMPLEX | 8 | 1.59 | 1.35E-02 | 9.71E-02 |
| REACTOME_SYNTHESIS_OF_VERY_LONG_CHAIN_FATTY_ACYL_COAS | 5 | 1.59 | 1.58E-02 | 9.75E-02 |
| MIPS_SNF2H_COHESIN_NURD_COMPLEX | 8 | 1.59 | 8.95E-03 | 9.85E-02 |
| REACTOME_TGF_BETA_RECEPTOR_SIGNALING_ACTIVATES_SMADS | 6 | 1.59 | 2.59E-02 | 1.01E-01 |
| REACTOME_HIV_LIFE_CYCLE | 45 | 1.58 | 0.00E+00 | 1.03E-01 |
| REACTOME_LATE_PHASE_OF_HIV_LIFE_CYCLE | 39 | 1.58 | 0.00E+00 | 1.03E-01 |
| REACTOME_MHC_CLASS_II_ANTIGEN_PRESENTATION | 29 | 1.58 | 7.10E-03 | 1.04E-01 |
| MIPS_DNA_SYNTHESOME_COMPLEX | 6 | 1.57 | 1.18E-02 | 1.08E-01 |
| PID_AURORA_A_PATHWAY | 11 | 1.57 | 1.63E-02 | 1.09E-01 |
| MIPS_HDAC1_ASSOCIATED_CORE_COMPLEX_CII | 7 | 1.56 | 2.00E-02 | 1.13E-01 |
| REACTOME_DESTABILIZATION_OF_MRNA_BY_KSRP | 10 | 1.56 | 2.43E-02 | 1.14E-01 |
| REACTOME_REGULATION_OF_GLUCOKINASE_BY_GLUCOKINASE_REGULATORY_PROTEIN | 12 | 1.56 | 2.16E-02 | 1.15E-01 |
| REACTOME_G_ALPHA1213_SIGNALLING_EVENTS | 12 | 1.56 | 2.03E-02 | 1.15E-01 |
| MIPS_20S_PROTEASOME | 11 | 1.57 | 1.74E-02 | 1.15E-01 |
| KEGG_RNA_DEGRADATION | 27 | 1.56 | 7.09E-03 | 1.16E-01 |
| REACTOME_SYNTHESIS_OF_SUBSTRATES_IN_N_GLYCAN_BIOSYTHESIS | 6 | 1.56 | 2.28E-02 | 1.17E-01 |
| REACTOME_DNA_REPAIR | 35 | 1.55 | 5.03E-03 | 1.20E-01 |
| MIPS_HCF_1_COMPLEX | 13 | 1.55 | 2.38E-02 | 1.21E-01 |
| REACTOME_TRANSPORT_OF_RIBONUCLEOPROTEINS_INTO_THE_HOST_NUCLEUS | 12 | 1.55 | 1.73E-02 | 1.22E-01 |
| MIPS_H2AX_COMPLEX_II | 5 | 1.55 | 2.95E-02 | 1.24E-01 |
| REACTOME_PREFOLDIN_MEDIATED_TRANSFER_OF_SUBSTRATE_TO_CCT_TRIC | 13 | 1.55 | 1.81E-02 | 1.25E-01 |
| REACTOME_PURINE_RIBONUCLEOSIDE_MONOPHOSPHATE_BIOSYNTHESIS | 7 | 1.54 | 2.45E-02 | 1.31E-01 |
| PID_LKB1_PATHWAY | 13 | 1.54 | 2.47E-02 | 1.31E-01 |
| REACTOME_GLUCOSE_TRANSPORT | 16 | 1.53 | 1.99E-02 | 1.34E-01 |
| REACTOME_TRANSPORT_OF_MATURE_MRNA_DERIVED_FROM_AN_INTRONLESS_TRANSCRIPT | 18 | 1.53 | 1.34E-02 | 1.35E-01 |
| MIPS_CEN_COMPLEX | 14 | 1.53 | 2.32E-02 | 1.43E-01 |
| REACTOME_RNA_POL_II_TRANSCRIPTION_PRE_INITIATION_AND_PROMOTER_OPENING | 14 | 1.52 | 2.95E-02 | 1.47E-01 |
| KEGG_MISMATCH_REPAIR | 8 | 1.51 | 2.96E-02 | 1.57E-01 |
| MIPS_ANTI_HDAC2_COMPLEX | 6 | 1.51 | 2.66E-02 | 1.57E-01 |
| REACTOME_MICRORNA_MIRNA_BIOGENESIS | 7 | 1.51 | 4.52E-02 | 1.58E-01 |
| REACTOME_MEIOTIC_SYNAPSIS | 7 | 1.51 | 3.55E-02 | 1.61E-01 |
| REACTOME_ASPARAGINE_N_LINKED_GLYCOSYLATION | 31 | 1.51 | 9.04E-03 | 1.62E-01 |
| KEGG_N_GLYCAN_BIOSYNTHESIS | 20 | 1.50 | 1.85E-02 | 1.67E-01 |
| REACTOME_REGULATORY_RNA_PATHWAYS | 7 | 1.49 | 3.02E-02 | 1.76E-01 |
| REACTOME_RNA_POL_II_TRANSCRIPTION | 40 | 1.49 | 7.00E-03 | 1.77E-01 |
| REACTOME_PROTEIN_FOLDING | 19 | 1.49 | 2.66E-02 | 1.82E-01 |
| REACTOME_FORMATION_OF_TUBULIN_FOLDING_INTERMEDIATES_BY_CCT_TRIC | 8 | 1.49 | 3.55E-02 | 1.82E-01 |
| MIPS_RC_COMPLEX_DURING_S_PHASE_OF_CELL_CYCLE | 7 | 1.48 | 3.53E-02 | 1.89E-01 |
| KEGG_BASE_EXCISION_REPAIR | 12 | 1.48 | 3.59E-02 | 1.91E-01 |
| REACTOME_POL_SWITCHING | 7 | 1.48 | 3.94E-02 | 1.91E-01 |
| REACTOME_TRANSCRIPTION | 59 | 1.47 | 6.01E-03 | 1.97E-01 |
| REACTOME_G0_AND_EARLY_G1 | 6 | 1.47 | 3.46E-02 | 1.97E-01 |
| KEGG_PROTEIN_EXPORT | 12 | 1.47 | 3.12E-02 | 1.97E-01 |
| KEGG_CELL_CYCLE | 39 | 1.47 | 1.00E-02 | 1.97E-01 |
| PID_E2F_PATHWAY | 23 | 1.47 | 2.24E-02 | 1.97E-01 |
| REACTOME_DEADENYLATION_DEPENDENT_MRNA_DECAY | 23 | 1.47 | 3.15E-02 | 1.98E-01 |
| REACTOME_FATTY_ACYL_COA_BIOSYNTHESIS | 7 | 1.47 | 3.60E-02 | 1.98E-01 |
| REACTOME_GLOBAL_GENOMIC_NER_GG_NER | 16 | 1.47 | 3.46E-02 | 2.00E-01 |
| REACTOME_LYSOSOME_VESICLE_BIOGENESIS | 9 | 1.47 | 4.12E-02 | 2.00E-01 |
| BIOCARTA_DEATH_PATHWAY | 6 | 1.46 | 4.19E-02 | 2.13E-01 |
| MIPS_KINASE_MATURATION_COMPLEX_1 | 8 | 1.45 | 5.51E-02 | 2.15E-01 |
| KEGG_NEUROTROPHIN_SIGNALING_PATHWAY | 25 | 1.45 | 2.94E-02 | 2.15E-01 |
| MIPS_MLL1_WDR5_COMPLEX | 10 | 1.45 | 4.23E-02 | 2.16E-01 |
| REACTOME_REPAIR_SYNTHESIS_FOR_GAP_FILLING_BY_DNA_POL_IN_TC_NER | 7 | 1.45 | 6.15E-02 | 2.17E-01 |
| MIPS_RC_COMPLEX_DURING_G2_M_PHASE_OF_CELL_CYCLE | 7 | 1.45 | 5.93E-02 | 2.20E-01 |
| REACTOME_ANTIVIRAL_MECHANISM_BY_IFN_STIMULATED_GENES | 25 | 1.45 | 3.04E-02 | 2.22E-01 |
| REACTOME_RNA_POL_II_PRE_TRANSCRIPTION_EVENTS | 23 | 1.44 | 3.67E-02 | 2.32E-01 |
| PID_P38_MK2PATHWAY | 6 | 1.44 | 5.65E-02 | 2.32E-01 |
| REACTOME_BIOSYNTHESIS_OF_THE_N_GLYCAN_PRECURSOR_DOLICHOL_LIPID_LINKED_OLIGOSACCHARIDE_LLO_AND_TRANSFER_TO_A_NASCENT_PROTEIN | 11 | 1.43 | 6.21E-02 | 2.36E-01 |
| BIOCARTA_CASPASE_PATHWAY | 7 | 1.43 | 5.83E-02 | 2.37E-01 |
| REACTOME_FORMATION_OF_TRANSCRIPTION_COUPLED_NER_TC_NER_REPAIR_COMPLEX | 13 | 1.43 | 4.70E-02 | 2.43E-01 |
| KEGG_BASAL_TRANSCRIPTION_FACTORS | 9 | 1.43 | 4.85E-02 | 2.44E-01 |
| REACTOME_THE_ROLE_OF_NEF_IN_HIV1_REPLICATION_AND_DISEASE_PATHOGENESIS | 10 | 1.43 | 6.33E-02 | 2.44E-01 |
| MIPS_RNA_POLYMERASE_II_HOLOENZYME_COMPLEX | 10 | 1.43 | 5.18E-02 | 2.44E-01 |
| REACTOME_SIGNALING_BY_RHO_GTPASES | 15 | 1.43 | 4.60E-02 | 2.45E-01 |
| REACTOME_MEIOTIC_RECOMBINATION | 7 | 1.43 | 5.10E-02 | 2.46E-01 |
| PID_FOXOPATHWAY | 16 | 1.42 | 4.64E-02 | 2.48E-01 |
| MIPS_LARGE_DROSHA_COMPLEX | 10 | 1.42 | 7.14E-02 | 2.49E-01 |
| REACTOME_RNA_POL_I_RNA_POL_III_AND_MITOCHONDRIAL_TRANSCRIPTION | 25 | 1.42 | 2.76E-02 | 2.50E-01 |

**Downregulated canonical pathways in *Myc*/*xmrk-*induced liver tumors**

| NAME | SIZE | NES | p-val | FDR |
| --- | --- | --- | --- | --- |
| KEGG_PEROXISOME | 32 | -3.46 | 0.00E+00 | 0.00E+00 |
| KEGG_FATTY_ACID_METABOLISM | 21 | -3.01 | 0.00E+00 | 0.00E+00 |
| REACTOME_PEPTIDE_CHAIN_ELONGATION | 66 | -2.90 | 0.00E+00 | 0.00E+00 |
| BIOCARTA_INTRINSIC_PATHWAY | 8 | -2.88 | 0.00E+00 | 0.00E+00 |
| REACTOME_PEROXISOMAL_LIPID_METABOLISM | 10 | -2.79 | 0.00E+00 | 0.00E+00 |
| KEGG_COMPLEMENT_AND_COAGULATION_CASCADES | 23 | -2.72 | 0.00E+00 | 0.00E+00 |
| KEGG_RETINOL_METABOLISM | 7 | -2.63 | 0.00E+00 | 0.00E+00 |
| KEGG_PPAR_SIGNALING_PATHWAY | 21 | -2.59 | 0.00E+00 | 0.00E+00 |
| REACTOME_BILE_ACID_AND_BILE_SALT_METABOLISM | 6 | -2.48 | 0.00E+00 | 9.09E-04 |
| MIPS_60S_RIBOSOMAL_SUBUNIT_CYTOPLASMIC | 39 | -1.87 | 0.00E+00 | 1.05E-03 |
| REACTOME_SYNTHESIS_OF_BILE_ACIDS_AND_BILE_SALTS | 6 | -2.39 | 0.00E+00 | 1.68E-03 |
| BIOCARTA_AMI_PATHWAY | 8 | -2.33 | 9.01E-03 | 2.85E-03 |
| BIOCARTA_EXTRINSIC_PATHWAY | 8 | -2.25 | 0.00E+00 | 4.14E-03 |
| KEGG_PRIMARY_BILE_ACID_BIOSYNTHESIS | 5 | -2.26 | 0.00E+00 | 4.30E-03 |
| REACTOME_SYNTHESIS_OF_BILE_ACIDS_AND_BILE_SALTS_VIA_7ALPHA_HYDROXYCHOLESTEROL | 5 | -2.27 | 0.00E+00 | 4.35E-03 |
| KEGG_TRYPTOPHAN_METABOLISM | 19 | -2.23 | 0.00E+00 | 4.92E-03 |
| REACTOME_GAMMA_CARBOXYLATION_TRANSPORT_AND_AMINO_TERMINAL_CLEAVAGE_OF_PROTEINS | 5 | -2.21 | 0.00E+00 | 5.45E-03 |
| PID_INTEGRIN2_PATHWAY | 7 | -2.13 | 0.00E+00 | 1.03E-02 |
| REACTOME_FORMATION_OF_FIBRIN_CLOT_CLOTTING_CASCADE | 13 | -2.10 | 0.00E+00 | 1.29E-02 |
| KEGG_MATURITY_ONSET_DIABETES_OF_THE_YOUNG | 10 | -2.09 | 0.00E+00 | 1.32E-02 |
| BIOCARTA_LONGEVITY_PATHWAY | 5 | -2.08 | 0.00E+00 | 1.36E-02 |
| REACTOME_COMMON_PATHWAY | 7 | -2.05 | 0.00E+00 | 1.50E-02 |
| REACTOME_REGULATION_OF_INSULIN_LIKE_GROWTH_FACTOR_IGF_ACTIVITY_BY_INSULIN_LIKE_GROWTH_FACTOR_BINDING_PROTEINS_IGFBPS | 5 | -2.01 | 0.00E+00 | 2.01E-02 |
| KEGG_CITRATE_CYCLE_TCA_CYCLE | 21 | -1.91 | 3.45E-02 | 3.21E-02 |
| KEGG_METABOLISM_OF_XENOBIOTICS_BY_CYTOCHROME_P450 | 9 | -1.91 | 1.05E-02 | 3.30E-02 |
| KEGG_DRUG_METABOLISM_CYTOCHROME_P450 | 9 | -1.86 | 1.12E-02 | 3.75E-02 |
| PID_HNF3BPATHWAY | 25 | -1.86 | 0.00E+00 | 3.89E-02 |
| REACTOME_BIOLOGICAL_OXIDATIONS | 28 | -1.86 | 0.00E+00 | 4.03E-02 |
| REACTOME_MITOCHONDRIAL_FATTY_ACID_BETA_OXIDATION | 10 | -1.84 | 0.00E+00 | 4.06E-02 |
| KEGG_BETA_ALANINE_METABOLISM | 10 | -1.84 | 1.19E-02 | 4.14E-02 |
| REACTOME_ABC_FAMILY_PROTEINS_MEDIATED_TRANSPORT | 5 | -1.81 | 9.30E-03 | 4.51E-02 |
| KEGG_PANTOTHENATE_AND_COA_BIOSYNTHESIS | 7 | -1.81 | 0.00E+00 | 4.62E-02 |
| KEGG_NEUROACTIVE_LIGAND_RECEPTOR_INTERACTION | 12 | -1.78 | 0.00E+00 | 5.41E-02 |
| KEGG_LIMONENE_AND_PINENE_DEGRADATION | 6 | -1.76 | 5.75E-03 | 5.79E-02 |
| REACTOME_RESPIRATORY_ELECTRON_TRANSPORT_ATP_SYNTHESIS_BY_CHEMIOSMOTIC_COUPLING_AND_HEAT_PRODUCTION_BY_UNCOUPLING_PROTEINS_ | 50 | -1.75 | 0.00E+00 | 5.96E-02 |
| REACTOME_PHASE1_FUNCTIONALIZATION_OF_COMPOUNDS | 10 | -1.73 | 1.15E-02 | 6.44E-02 |
| KEGG_GLYCOLYSIS_GLUCONEOGENESIS | 24 | -1.70 | 0.00E+00 | 7.38E-02 |
| KEGG_BIOSYNTHESIS_OF_UNSATURATED_FATTY_ACIDS | 9 | -1.69 | 9.90E-03 | 7.69E-02 |
| REACTOME_COMPLEMENT_CASCADE | 9 | -1.67 | 1.09E-02 | 8.32E-02 |
| REACTOME_ALPHA_LINOLENIC_ACID_ALA_METABOLISM | 6 | -1.63 | 4.17E-02 | 1.06E-01 |
| REACTOME_RIP_MEDIATED_NFKB_ACTIVATION_VIA_DAI | 5 | -1.60 | 2.00E-02 | 1.19E-01 |
| KEGG_CELL_ADHESION_MOLECULES_CAMS | 13 | -1.59 | 3.33E-02 | 1.25E-01 |
| REACTOME_TAK1_ACTIVATES_NFKB_BY_PHOSPHORYLATION_AND_ACTIVATION_OF_IKKS_COMPLEX | 5 | -1.56 | 4.09E-02 | 1.40E-01 |
| REACTOME_LIPID_DIGESTION_MOBILIZATION_AND_TRANSPORT | 8 | -1.55 | 8.93E-03 | 1.47E-01 |
| KEGG_GLYCINE_SERINE_AND_THREONINE_METABOLISM | 18 | -1.53 | 2.63E-02 | 1.58E-01 |
| BIOCARTA_NDKDYNAMIN_PATHWAY | 5 | -1.52 | 7.18E-02 | 1.61E-01 |
| KEGG_ALPHA_LINOLENIC_ACID_METABOLISM | 6 | -1.51 | 5.36E-02 | 1.63E-01 |
| REACTOME_REGULATION_OF_BETA_CELL_DEVELOPMENT | 10 | -1.50 | 7.87E-02 | 1.64E-01 |
| KEGG_ASCORBATE_AND_ALDARATE_METABOLISM | 5 | -1.51 | 5.76E-02 | 1.65E-01 |
| KEGG_VALINE_LEUCINE_AND_ISOLEUCINE_DEGRADATION | 26 | -1.50 | 0.00E+00 | 1.67E-01 |
| MIPS_TRBP_CONTAINING_COMPLEX_1 | 21 | -1.49 | 9.09E-02 | 1.67E-01 |
| REACTOME_SYNTHESIS_OF_PIPS_AT_THE_EARLY_ENDOSOME_MEMBRANE | 5 | -1.48 | 5.61E-02 | 1.69E-01 |
| REACTOME_CLASS_A1_RHODOPSIN_LIKE_RECEPTORS | 12 | -1.48 | 7.69E-02 | 1.72E-01 |
| REACTOME_SYNTHESIS_OF_PIPS_AT_THE_GOLGI_MEMBRANE | 5 | -1.46 | 8.82E-02 | 1.82E-01 |
| REACTOME_LIPOPROTEIN_METABOLISM | 6 | -1.45 | 6.36E-02 | 1.94E-01 |
| REACTOME_REGULATION_OF_GENE_EXPRESSION_IN_BETA_CELLS | 8 | -1.44 | 8.40E-02 | 1.95E-01 |
| KEGG_PROPANOATE_METABOLISM | 16 | -1.43 | 5.13E-02 | 1.97E-01 |
| BIOCARTA_GLYCOLYSIS_PATHWAY | 5 | -1.41 | 7.14E-02 | 2.09E-01 |
| PID_FGF_PATHWAY | 12 | -1.42 | 9.38E-02 | 2.11E-01 |
